# Redefining Therapies for Drug‐Resistant Tuberculosis: Synergistic Effects of Antimicrobial Peptides, Nanotechnology, and Computational Design

**DOI:** 10.1002/adhm.202503964

**Published:** 2026-01-19

**Authors:** Christian S. Carnero Canales, Jessica Ingrid Marquez Cazorla, Renzo Marianito Marquez Cazorla, Aline Martins dos Santos, Jonatas Lobato Duarte, Letícia Oliveira Catarin Nunes, Túlio Custódio Reis, Lara Cerazi Salvador, Norival Alves Santos‐Filho, Rafael Miguel Sábio, Hélder A. Santos, Fernando Rogério Pavan

**Affiliations:** ^1^ Vicerrectorado de Investigación Universidad Autónoma del Perú (UA) Lima Perú; ^2^ School of Pharmacy Biochemistry and Biotechnology Universidad Católica Santa María (UCSM) Arequipa Perú; ^3^ Nanovida Research Center Arequipa Perú; ^4^ School of Pharmaceutical Sciences São Paulo State University Araraquara Brazil; ^5^ Institute of Chemistry São Paulo State University Araraquara Brazil; ^6^ Tuberculosis Research Laboratory School of Pharmaceutical Sciences São Paulo State University Araraquara Brazil; ^7^ Department of Biomaterials and Biomedical Technology The Personalized Medicine Research Institute (PRECISION) University Medical Center Groningen University of Groningen Groningen The Netherlands

**Keywords:** antimicrobial peptides, drug delivery systems, drug discovery, mycobacterium species, tuberculosis

## Abstract

Tuberculosis (TB), caused by *Mycobacterium tuberculosis* (*Mtb*), remains a major global health concern, particularly due to the emergence of multidrug‐resistant and extensively drug‐resistant strains. The persistence and propagation of TB are favored by the pathogen's sophisticated virulence mechanisms, its ability to evade immune responses, and the formation of latent infections within granulomas. Current therapeutic regimens are limited by long treatment durations, drug resistance, and significant socioeconomic burdens. Antimicrobial peptides (AMPs) have emerged as promising alternatives because of their broad‐spectrum activity and reduced likelihood of resistance development. Nevertheless, their clinical application is hindered by rapid proteolytic degradation, low specificity and limited bioavailability. Recent advances in nanotechnology have facilitated the encapsulation and targeted delivery of AMPs, improving their therapeutic potential against TB. Furthermore, the integration of computational approaches—such as molecular docking and molecular dynamics (MD) simulations—has enabled the rational design and optimization of AMPs, expediting the discovery of novel anti‐TB agents. This review summarizes the pathogenesis and resistance mechanisms of *Mtb*, highlights the current landscape and limitations of AMP‐based therapies, and discusses the role of nanotechnology and in silico tools in the development of new treatment strategies for TB.

## Graphical abstract

Antimicrobial peptide (AMP)‐loaded nanocarriers provide a multifunctional strategy to combat drug‐resistant Mycobacterium tuberculosis. By enhancing intracellular delivery, bypassing efflux pumps, and disrupting bacterial membranes, this platform restores phagolysosome fusion and macrophage function. The synergistic action results in efficient bacterial killing, reduced inflammation, and sustained clearance of resistant tuberculosis infection.

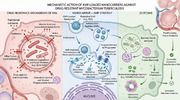

## 1 Introduction

Tuberculosis (TB), an infectious disease that primarily affects the pulmonary system, is caused by *Mycobacterium tuberculosis* (*Mtb*) but can also disseminate to other organs [1]. Transmission predominantly occurs via aerosolized droplets generated through speech, coughing, or sneezing by infected individuals [2]. Although TB is one of humanity's oldest diseases, it continues to represent a substantial public health burden, particularly in regions with fragile healthcare infrastructures. Standard therapy comprises prolonged, multidrug antibiotic regimens, which are essential for achieving complete bacterial clearance [3, 4]. Effective TB control is contingent on prompt diagnosis, reliable access to efficacious drugs, and the implementation of preventive measures, underscoring the importance of both community awareness and robust healthcare delivery systems in limiting its prevalence worldwide [5].

A major setback in global TB control efforts arose during the COVID‐19 pandemic, reversing the decade‐long trend of declining incidence rates. Notably, between 2020 and 2022, the incidence of TB rose by 3.9%, with the number of affected individuals increasing from 10 million in 2020 to 10.6 million in 2022—contrasting sharply with the previous annual reduction of nearly 2% [6, 7, 8]. This reversal is attributed largely to pandemic‐driven disruptions in essential health services. Nonetheless, sustained interventions contributed to a decrease in TB‐related mortality, which decreased from 1.42 million in 2021 to 1.25 million in 2023, reflecting persistent efforts to mitigate the impact of TB despite the ongoing global health crisis [9, 10].

As drug resistance becomes increasingly prevalent, antimicrobial peptides (AMPs) are attracting attention as promising alternatives for TB therapy because of their broad‐spectrum activity and limited potential for inducing resistance [11]. AMPs are naturally occurring or synthetic molecules that are generally composed of 10 to 50 amino acids and can display a wide range of secondary structures, including α‐helices, β‐sheets, or extended conformations [12]. This structural diversity enables them to interact selectively with microbial membranes, often causing rapid cell lysis, and to influence host immune pathways by modulating inflammatory responses and enhancing pathogen clearance. Their versatility and unique mechanisms make them especially valuable in the context of multidrug‐resistant TB [13]. Moreover, AMPs can be isolated from various natural sources—such as animals, plants, and microorganisms—or designed de novo to optimize pharmacological profiles and minimize toxicity [14].

Traditional antitubercular peptide discovery is often labor intensive and slow. Advances in computational methods have accelerated this process, with in silico approaches leveraging biological and chemical data to support structure‐based and ligand‐based drug design [12, 15]. These computational strategies facilitate the prediction of peptide activity, optimization of molecular interactions, and virtual screening of extensive compound libraries [16]. The integration of artificial intelligence and machine learning algorithms further enhances the identification and rational design of novel peptides or small molecules with optimized efficacy, stability, and safety profiles, thereby reducing the time and cost associated with conventional drug development [17]. In silico tools also allow for high‐throughput evaluation of potential off‐target effects, resistance profiles, and pharmacokinetic properties before experimental validation, contributing to more efficient and targeted development pipelines [18, 19].

**SCHEME 1:**
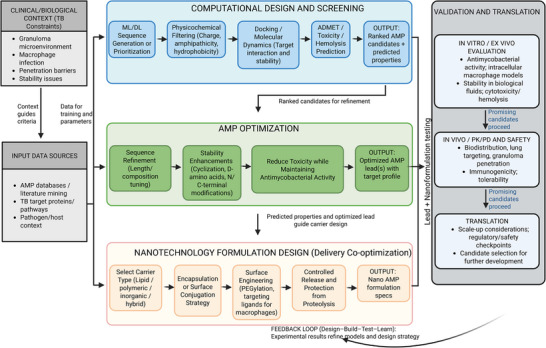
Integrated workflow for TB AMP discovery and nano‐delivery. This scheme illustrates a unified and iterative pipeline that integrates tuberculosis‐specific biological constraints with computational design, AMP optimization, and nanotechnology‐based delivery. Clinical and biological context, together with curated input data, guides in silico sequence generation, physicochemical filtering, molecular docking, molecular dynamics, and toxicity prediction to prioritize AMP candidates. Optimized peptides are subsequently co‐designed with nanocarrier systems to enhance stability, targeting, and controlled release, followed by in vitro/ex vivo and in vivo validation and translational assessment. Experimental outcomes feed back into computational and formulation stages through a Design‐Build‐Test‐Learn loop, enabling continuous refinement toward clinically relevant anti‐tuberculosis nano‐therapeutics.

Research efforts have increasingly focused on nanotechnology to address these obstacles. Nanocarrier‐based delivery systems protect peptides from enzymatic degradation, improve their solubility and bioavailability, and allow sustained, site‐directed release at infection foci [20, 21]. These nanosystems can be designed with various materials—including polymers, lipids, and inorganic matrices—to optimize the physicochemical properties and therapeutic indices of encapsulated AMPs [22]. Additionally, surface functionalization strategies, such as peptide conjugation or ligand attachment, facilitate targeted delivery to specific cells or tissues, minimizing off‐target effects and systemic toxicity [23]. To provide an integrated view of how AMPs, computational design, and nanotechnology operate together in TB therapy development, we propose the unified, iterative workflow shown in Scheme 1.

## 2 Tuberculosis

### 2.1 Mycobacterium Tuberculosis (*Mtb*)

*Mtb* is the etiological agent of human tuberculosis and a highly adapted pathogen that deploys multiple virulence determinants to persist in the host despite mucosal defenses and antibiotic pressure [24]. It is a slow‐growing, acid‐fast bacillus with a lipid‐rich cell envelope and a facultative intracellular lifestyle, features that complicate macrophage‐mediated clearance [25].

Macrophages are among the first cellular targets and strongly shape early disease outcome. They may constrain bacterial replication or, conversely, provide a permissive intracellular niche that supports proliferation and spread within lung tissue, contributing to granuloma development [26]. Infection is initiated after inhalation of aerosolized bacilli, followed by transit through the airways to the distal lung and deposition in the alveolar spaces [27].

During early infection, alveolar macrophages and dendritic cells serve as initial reservoirs and promote the subsequent activation of adaptive immunity, including T‐ and B‐lymphocyte responses [28]. Together with recruited inflammatory monocytes, neutrophils, and additional lymphocyte populations driven by inflammation and tissue injury, these cells organize into the characteristic tuberculous granuloma [29, 30]. Granuloma formation helps limit bacterial dissemination and favors a latent state, resulting in latent TB infection (LTBI), which can persist for years before progressing to active disease [31]. An estimated 5–10% of individuals with LTBI will develop active TB [32], with substantially higher risk in immunocompromised settings, such as HIV/AIDS, diabetes, and viral coinfections [33].

**FIGURE 1:**
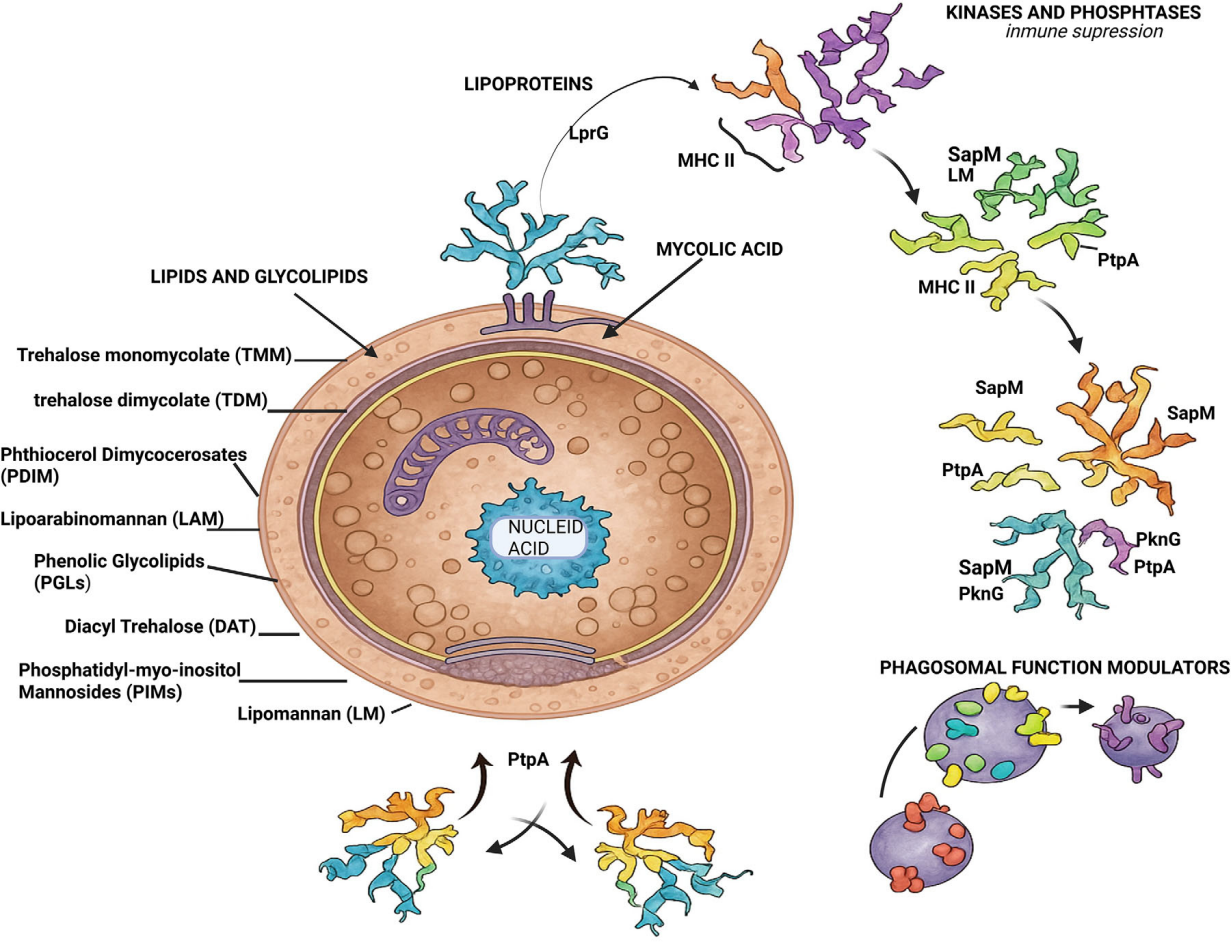
Schematic representation of the principal proteolytic and nonproviral virulence factors of *Mtb*, including their location in the cell envelope and their roles in immune evasion and intracellular survival.

*Mtb* persistence is closely linked to its ability to reshape innate immune programs through both protein and non‐protein virulence determinants that support immune evasion, intracellular survival, and dissemination [34]. The principal determinants discussed here are summarized in Table 1, and their overall localization and routes of action are outlined in Figure 1. Broadly, envelope‐associated lipids and glycoconjugates influence permeability and immune recognition, whereas protein determinants, including secreted effectors and surface‐associated factors, disrupt processes, such as phagosome maturation, host signaling, and antigen presentation [35].

**TABLE 1:** Principal virulence factors of *Mtb* and their roles.

Type	Virulence factor	Role/Mechanism	References
**Nonproteic**	Mycolic acids, TDM, TMM	Increase cell envelope impermeability; inhibit phagosome maturation and immune recognition	[36, 37, 38, 39, 40]
**Nonproteic**	PDIM, PGL, DAT	Evade TLR detection; suppress immune signaling; recruit infected macrophages; inhibit inflammatory cytokines	[35, 41, 42, 43, 44]
**Nonproteic**	PIMs, LM, LAM	Block phagolysosome fusion; modulate TLR and cytokine responses; create intracellular survival niche	[45, 46, 47]
**Nonproteic**	Peptidoglycan, arabinogalactan	Provide cell wall rigidity and resistance to host defenses	[48]
**Nonproteic**	DNA (via ESX/type VII secretion)	Activates cGAS/STING pathway, modulating immunity and inducing autophagy	[49]
**Proteic**	LprG (lipoprotein)	Modulates antigen presentation; interferes with TLR signaling; affects immune response	[50]
**Proteic**	PknG (kinase), SapM and PtpA (phosphatases)	Inhibit phagosome maturation; suppress macrophage activity; promote intracellular persistence	[51, 52]
**Proteic**	ESX secretion system (including effectors)	Secretes proteins that manipulate host cell functions; essential for pathogenicity	[53]

Abbreviations: Trehalose dimycolate (TDM); Trehalose monomycolate (TMM); Phthiocerol dimycocerosates (PDIM); Phenolic glycolipids (PGL); Diacyl trehalose (DAT); Phosphatidyl‐myo‐inositol mannosides (PIMs); Lipomannan (LM); Lipoarabinomannan (LAM); Lipoprotein G (LprG); Protein kinase G (PknG); Secreted acid phosphatase (SapM); Protein tyrosine phosphatase A (PtpA); ESX secretion system (type VII secretion system); Toll‐like receptor (TLR); Cyclic GMP‐AMP synthase (cGAS); Stimulator of interferon genes (STING).

### 2.2 Resistance Mechanisms

**FIGURE 2:**
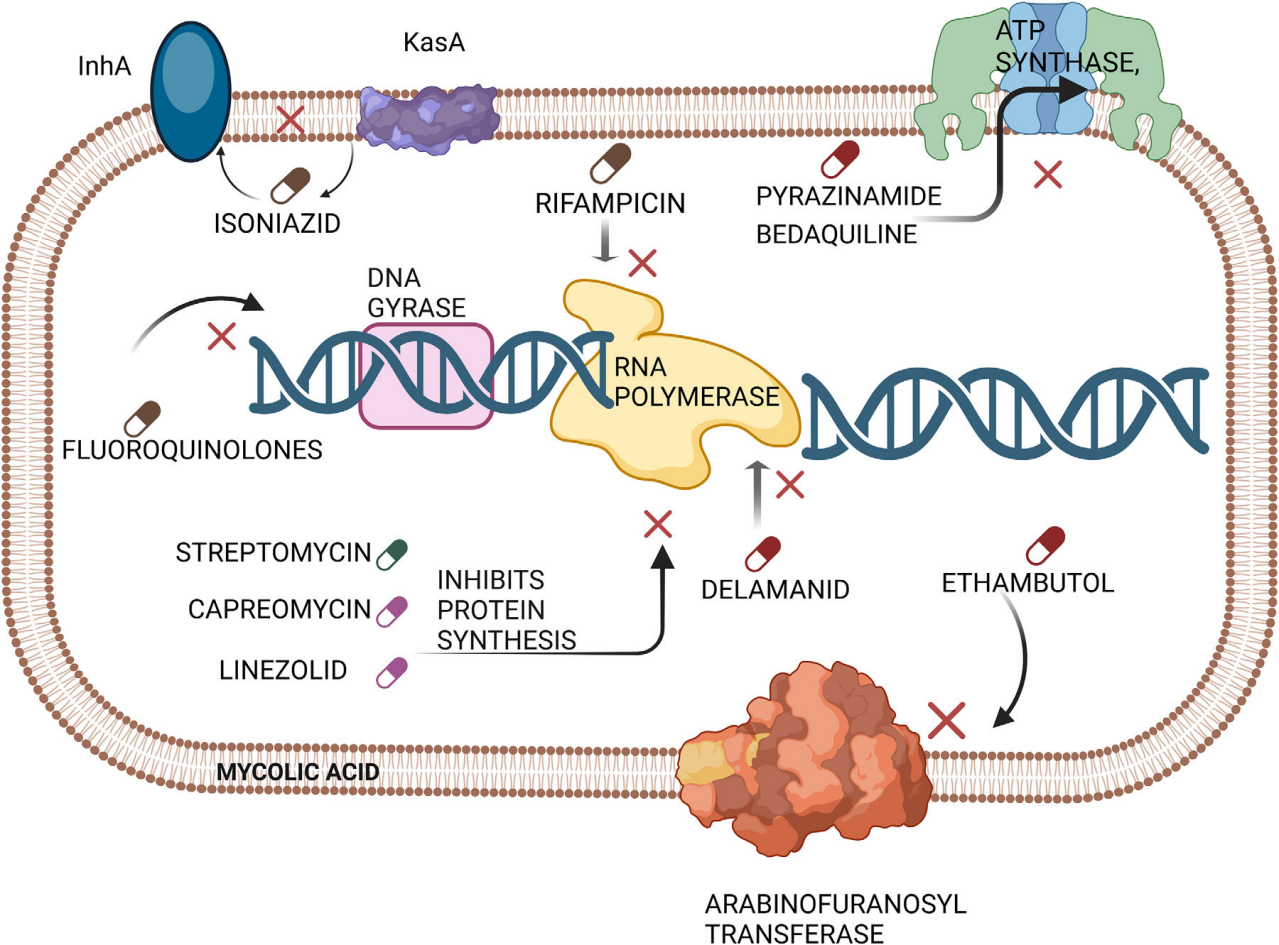
Schematic representation of the cellular targets of first‐ and second‐line antibiotics used in TB treatment, including inhibitors of cell wall biosynthesis (INH, ethambutol, delamanid), protein synthesis (streptomycin, capreomycin, linezolid), DNA gyrase (fluoroquinolones), RNA polymerase (RIF), and ATP synthase (bedaquiline). The figure also highlights how *Mtb* can develop resistance through mutations in target genes, decreased cell wall permeability, and other molecular mechanisms.

Currently used antibiotics against *Mtb* mainly target cell wall biosynthesis, protein synthesis, nucleotide synthesis, lipid metabolism, and RNA polymerase inhibition (Figure 2). *Mtb*, through its diverse virulence factors, has developed resistance mechanisms against these drugs [54, 55]. TB treatment relies on therapeutic regimens encompassing different generations of antibiotics. First‐generation drugs, including isoniazid (INH), rifampicin (RIF), pyrazinamide, ethambutol, and streptomycin, constitute the foundation of antibiotic regimens [56, 57]. These drugs employ various mechanisms of action: INH is a prodrug that is enzymatically activated by catalase (KatG) and subsequently inhibits the synthesis of mycolic acid, a vital component of the *Mtb* cell wall. RIF binds to RNA polymerase, blocking bacterial gene expression; pyrazinamide, which is also a prodrug, is activated by pyrazinamidase and is thought to disrupt membrane potential and reduce ATP synthesis; and ethambutol inhibits the synthesis and polymerization of arabinan by blocking arabinosyl transferase, thus compromising cell wall biosynthesis [58].

Genetic studies of *Mtb* have identified mutations associated with acquired antibiotic resistance, mainly in the following genes: *rpoB* (RIF), *inhA* and *katG* (INH), and *gyrA* (fluoroquinolones such as levofloxacin and moxifloxacin) [59]. Among the multiple resistance mechanisms developed by *Mtb*, several molecular mechanisms stand out: decreased cell wall permeability reduces antibiotic transport; efflux pumps expel antibiotics; and alterations in cellular metabolism and genetic mutations confer further resistance [60].

Second‐generation antibiotics include aminoglycosides such as amikacin, kanamycin, and capreomycin, which are resistant because of mutations in the *rrs* gene. Additionally, fluoroquinolones (e.g., ciprofloxacin) are resistant to mutations in the *gyrA* and *gyrB* genes, which encode DNA gyrases. Para‐aminosalicylic acid acts by competing with PABA for dihydropteroate synthase, inhibiting folate synthesis, with resistance arising from mutations in the *thyA* gene. Ethionamide, a prodrug activated by the monooxygenase encoded by *ethA*, inhibits mycolic acid synthesis, with resistance resulting from mutations in *ethA* and *inhA* [61]. Despite advances in treatment, *Mtb* grows slowly in conventional media, and antibiotic susceptibility testing is time‐consuming; phenotypic sensitivity tests may take up to eight weeks, delaying treatment and increasing resistance risk [62]. Molecular assays, therefore, aid in detecting resistance to antibiotics such as RIF, INH, and fluoroquinolones, as well as identifying mutations and facilitating bacterial genome sequencing [63].

The WHO classifies TB resistance into five categories: monoresistance (resistance to one first‐line drug), polyresistance, multidrug resistance (MDR; resistance to at least RIF and INH), extensively drug‐resistant TB (XDR; resistance to any fluoroquinolone and at least one of three second‐line injectable drugs), and RIF resistance (RR) [64]. Globally, INH resistance without concomitant RIF resistance is the most common form of resistance, accounting for approximately 10.6% of TB cases [65]. MDR‐TB refers to *Mtb* strains resistant to the first‐line agents INH and RIF, whereas XDR‐TB includes further resistance. Notably, most XDR‐TB cases arise from new mutations acquired during MDR‐TB treatment rather than primary importation of XDR strains [64, 66].

### 2.3 Epidemiology

TB is predominantly associated with low‐ and middle‐income countries, where the incidence reaches approximately 183 cases per 100 000 people per year, whereas it is less than 10 cases per 100 000 per year in high‐income countries [67]. This widely disseminated infection was the leading cause of death from infectious diseases in 2023, surpassing HIV and COVID‐19. Despite governmental and international efforts, a substantial proportion of cases remain undiagnosed, including an estimated 275 000 patients with drug‐resistant TB who do not receive timely treatment [68]. Recent global prevalence studies have reported that the current prevalence of MDR‐TB is 11.6%, whereas XDR‐TB accounts for 2.5% of cases [69]. The COVID‐19 pandemic disrupted progress in the fight against MDR‐ and XDR‐TB, leading to an increase in new cases and mortality, as well as a reduction in the implementation of diagnostic testing and access to appropriate treatment. These setbacks have contributed to the worsening of antibiotic resistance [70]. Each year, more than 10 million new TB cases are reported globally, with approximately half a million exhibiting resistance to one or more drugs included in standard therapeutic regimens [71].

Antibiotic susceptibility testing is divided into phenotypic and genotypic methods, the latter of which have advanced considerably with techniques such as quantitative real‐time fluorescence PCR, GeneXpert *MTB*/RIF and GeneXpert‐Ultra, digital PCR, and next‐generation sequencing, all of which allow for the detection of resistance to several of the antibiotics currently in use [72].

In 2023, the estimated annual global number of patients who develop MDR‐TB or RR‐TB was approximately 400 000, with approximately 150 000 deaths attributed to these forms of TB—placing them among the leading causes of death from infectious diseases worldwide [9]. Treatment success rates improved from 50% in 2012 to 60% in 2019, largely due to the use of longer therapeutic regimens and the introduction of new antibiotics such as bedaquiline, pretomanid, and linezolid [73].

### 2.4 Complications with Current Treatment

Active TB can be fatal if not treated appropriately, with mortality rates reaching 50% [74]. Regimens for drug‐susceptible TB (DS‐TB) have already been standardized by the WHO in four guidelines that vary in complexity. As noted earlier, *Mtb* has the capacity to develop resistance due to its numerous virulence factors, making the cell wall the primary pharmacological target for drug design, although other targets include protein synthesis, energy metabolism, and nucleic acid synthesis and repair [75].

Currently, cases of drug‐susceptible TB are treated with three to four antibiotics for a period of six to nine months, whereas complicated cases of MDR‐TB may require seven to nine antibiotics in therapeutic regimens that can be extended for several additional months [76]. The duration of current therapeutic regimens ranges from six months for DS‐TB to up to eighteen months for XDR‐TB [77].

Another complication of MDR‐TB is the high cost of treatment, which often limits patient access. Even more concerning is its association with high mortality rates (up to 80%) resulting from inadequate or absent therapy. In this context, new drugs have been developed to optimize treatment, including bedaquiline, delamanid, and pretomanid. These drugs have gained prominence as promising options for reducing treatment duration and achieving high success rates (up to 89%) [78]. Importantly, treatment failure in DS‐TB can result from numerous factors, such as poor patient adherence, alcohol consumption, smoking, and coinfections. *Mtb* is listed among the WHO's priority‐resistant bacteria, and even with widely used therapeutic regimens, the cure rate for MDR‐TB and RR‐TB is 60%, whereas for XDR‐TB, it is 39% [79].

New therapeutic options for DS‐TB currently include a four‐month regimen of INH, rifapentine, moxifloxacin, and pyrazinamide, which has demonstrated noninferiority over the classic six‐month RIPE regimen in terms of sputum conversion and twelve‐month survival [80]. HIV‐positive patients represent a particularly vulnerable group, partly due to immune suppression and concomitant therapy, and TB remains the cause of 25% of all deaths worldwide in this population. Evidence indicates that newer regimens containing bedaquiline, pretomanid, linezolid, and moxifloxacin have improved outcomes and are recommended by the WHO and CDC as the current standard for resistant TB in both HIV‐positive and HIV‐negative individuals [81, 82]. Ongoing research is exploring nanotechnology‐based solutions for the treatment of MDR‐ and XDR‐TB. Nanoparticles offer several benefits, including enhanced antibiotic stability, improved absorption, controlled release, and targeted delivery to specific cells, all of which improve the efficacy and efficiency of antibiotic regimens [83].

## 3 Antimicrobial Peptides

The growing concern about antimicrobial resistance, as well as the side effects resulting from the uncontrolled use of antibiotics, for example, raises the alarm for the search for alternative treatments [84]. In this context, AMPs are emerging due to their activity, availability, and stability and the possibility of modifications that increase their activity [85, 86]. Nevertheless, understanding their mechanisms of action, as well as the challenges and limitations related to their use, is fundamental for understanding and directing molecules to specific targets and designing specific modifications [87, 88, 89].

### 3.1 Structure and Physicochemical Properties

AMPs are multifunctional therapeutic agents that can be classified according to their origin, method of synthesis, secondary structure and biological function [90, 91]. They have natural origins, such as from animals (such as amphibians, reptiles, mammals and insects), plants and bacteria, or synthetic origins, obtained by chemical, enzymatic or recombinant DNA synthesis [92, 93].

AMPs are also called “natural antibiotics” because of their broad spectrum of action against microorganisms, lower risk of resistance, because their mechanism of action is generally on the cell membrane, and ability to inhibit microorganisms that are resistant to conventional antibiotics, making AMPs ideal candidates for various pharmaceutical applications [94]. One of the main challenges in the application of AMPs derived from natural sources is their inherent limitations, such as low stability, susceptibility to proteolytic degradation, poor tolerance to pH and temperature variations, and high toxicity [95]. Studies linking peptide structures and antimicrobial activity have indicated that changes in physicochemical and structural parameters (such as net charge, hydrophobicity, secondary structure and solubility) can affect the activity observed [96, 97]. This discovery has enabled modifications to the amino acid sequence, mainly by rational design, to optimize the results achieved by broadening the spectrum of action and stability of the peptides [98].

Understanding the structural arrangement of AMPs is extremely important for understanding their mechanism of action and biological activities. Many methods can be used to investigate structure and activity, such as nuclear magnetic resonance (NMR) techniques, atomic force spectroscopy (AFM) and X‐ray crystallography combined with modeling, docking and MD [94]. Considering their structures, peptides can be classified into four main groups according to the observed secondary structure: α‐helix peptides, peptides with β‐sheets, αβ peptides and nonαβ peptides [99]. Most AMPs have an α‐helix conformation, which is generally stabilized by three factors: the interaction of hydrogen bonds between N─H and C═O, hydrophobic interactions, and electrostatic interactions of the side chains [100].

Most AMPs do not have a defined structure in aqueous solution but adopt an α‐helical conformation in membrane mimetic solutions, with hydrophobic residues on one side of the helix and hydrophilic residues on the other side, giving AMPs an amphipathic character [101]. However, it is not uncommon to observe other types of conformations in AMPs, such as cysteine‐containing cyclic peptides, which can be structured as β‐sheets in solution in the presence of one or more disulfide bonds [102]. In addition, triple β‐sheets, mixed α‐helix/β‐sheets, and disordered linear structures rich in glycine, proline, and arginine, for example, also act on the lipid membrane [103].

The structures and physicochemical properties of AMPs directly influence their biological activity [87]. However, in addition to the properties mentioned above, it is important to note that the amino acid sequence itself is extremely important for the performance of biological activity [97]. Changing just one residue can increase the cytotoxicity of the peptide or alter the hydrophobicity of the molecule and even increase the activity already observed [104]. The combination of these factors results in AMPs with different therapeutic targets, including antibacterial, antifungal, antiviral, antiparasitic, anticancer, and immunomodulatory properties.

### 3.2 Mechanisms of Action of AMPs

Several AMPs act through mechanisms beyond membrane destabilization, targeting essential intracellular processes. These include the inhibition of DNA, RNA, and protein synthesis; interference with enzymatic activity‐modifying enzymes; and disruption of cell wall formation. All these action mechanisms are discussed below [97].

#### 3.2.1 Membrane Interaction

The composition of AMPs is of fundamental importance to their interaction with the membrane because of their cationic nature, which interacts with negatively charged components found on the outer surface of the cell membrane, thus favoring cell lysis and the release of their intracellular contents [97]. The interaction between the peptide and the membrane is electrostatic in nature and does not depend on specific receptors in the membrane for binding and affinity. However, after binding to the anionic lipids present within the cell membrane, AMPs lose their disordered structure and conform to an α‐helix, facilitating their interaction with the membrane [105, 106].

**FIGURE 3:**
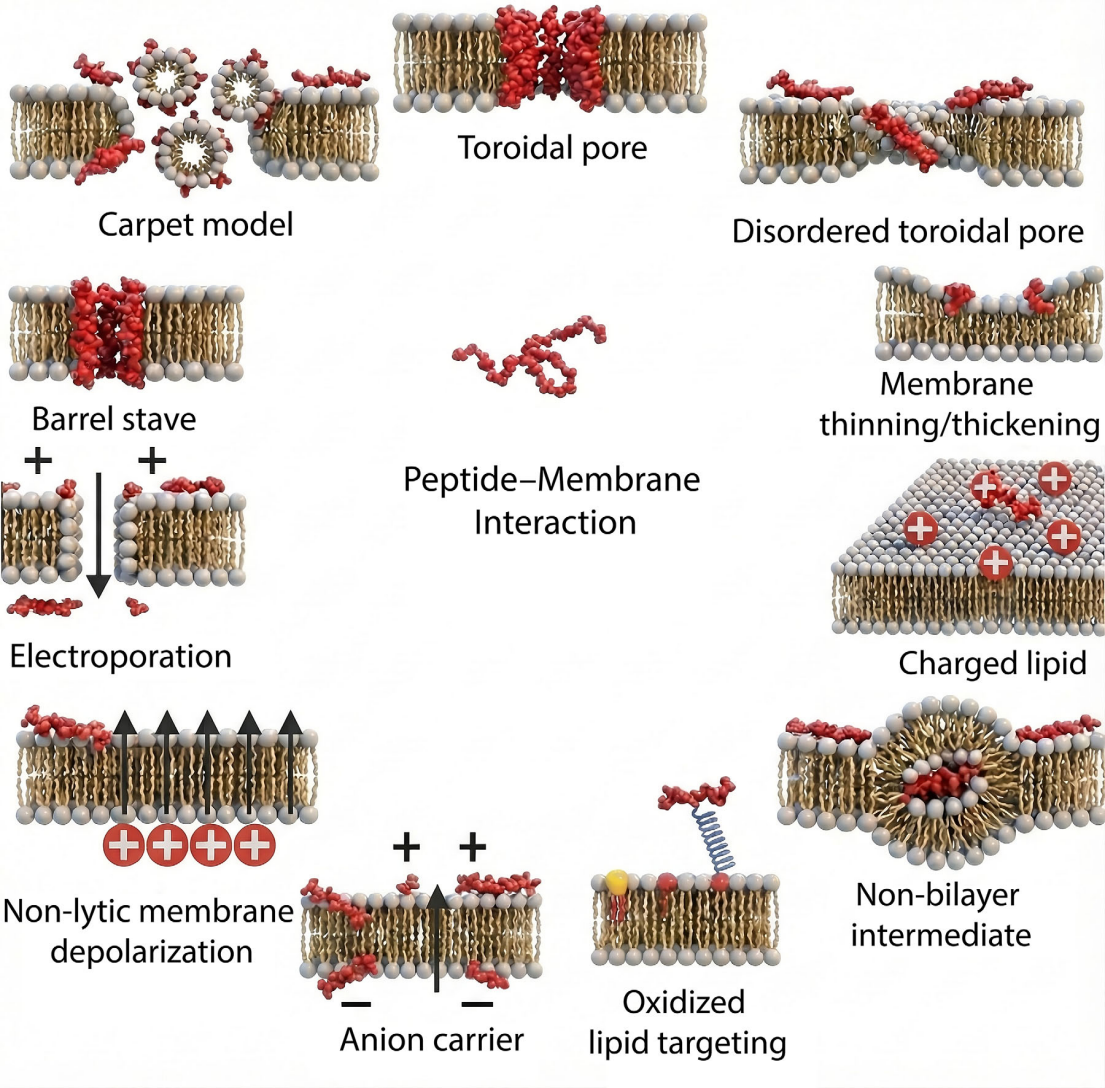
Mechanisms of peptide–membrane interaction and bacterial membrane disruption induced by antimicrobial peptides. The principal mechanisms by which AMPs compromise bacterial membrane integrity are presented, including the barrel stave, toroidal pore, and carpet models. Additional proposed mechanisms are also shown, such as disordered toroidal pore formation, electroporation, membrane thinning or thickening, non‐lytic membrane depolarization, anion carrier activity, clustering of charged lipids, formation of non‐bilayer intermediates, and targeting of oxidized lipids. Adapted from Carnero et al. [109], with permission from Elsevier (2025).

AMPs aggregate and reorient themselves in such a manner that they can lyse the cell membrane. This aggregation and its ability to affect membrane integrity are well described in the literature [107, 108]. AMPs act through different mechanisms (Figure 3), the main ones being the barrel stave model, the toroidal pore model, and the carpet model [109].

In the Barrel–Stave model, AMPs are inserted perpendicularly into the lipid bilayer, enabling the formation of transmembrane pores. This interaction is facilitated by the hydrophobic regions of the molecule, which bind to the membrane lipids, while the hydrophilic regions are responsible for lining the inside of the formed pore, allowing an uncontrolled flow of ions and molecules [110]. The toroidal pore model, which is similar to the barrel stave model, is distinguished by the induction of lipid curvature by peptides, resulting in the formation of pores coated with peptides and polar lipids [108]. In the carpet model, AMPs are arranged parallel to the surface of the cell membrane, covering it like a carpet. Disorganization of the lipid bilayer occurs at a critical concentration, leading to lysis without the formation of fully defined pores [111].

#### 3.2.2 Intracellular Mechanism

**FIGURE 4:**
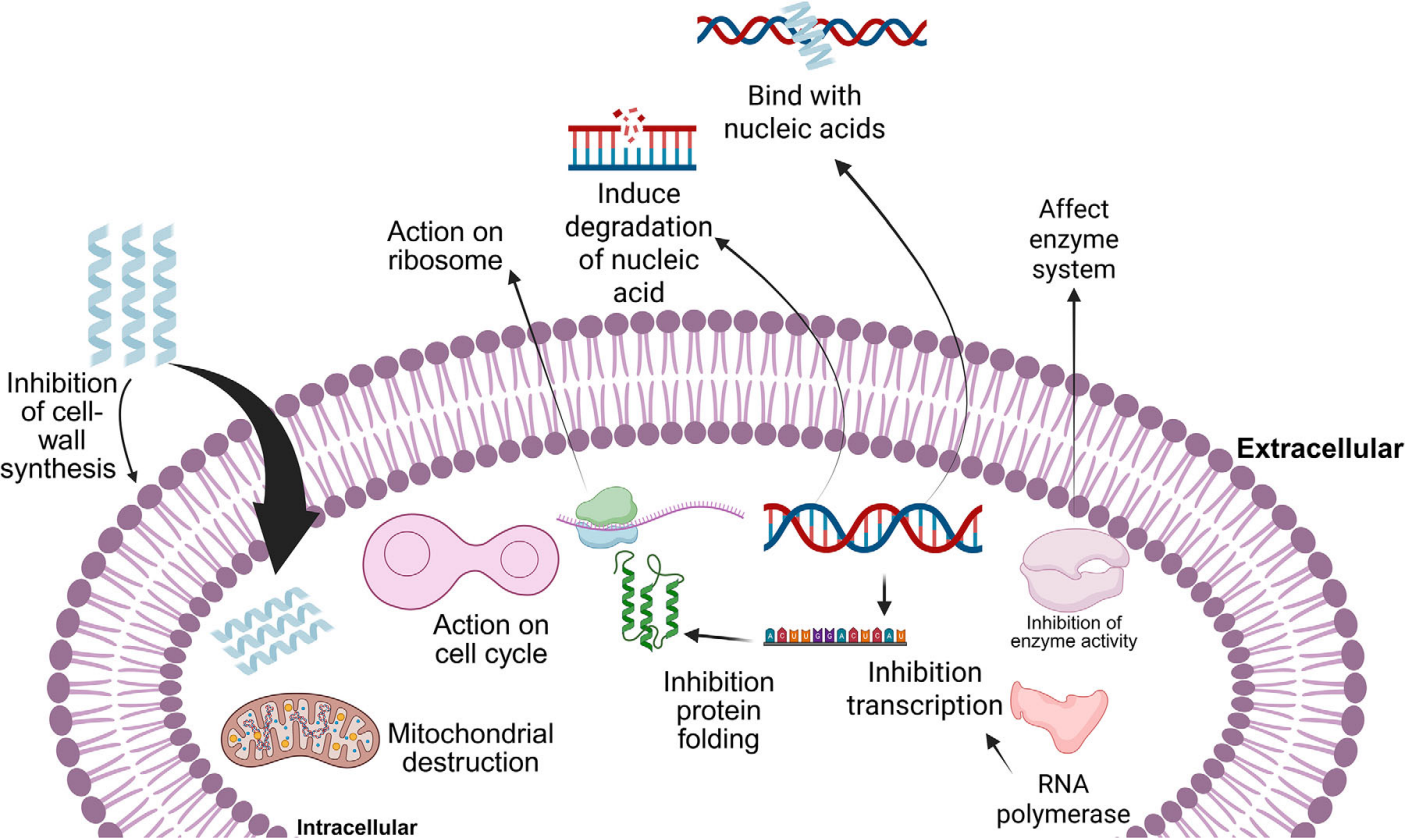
Intracellular targets of antimicrobial peptides following membrane translocation. After crossing the bacterial membrane, AMPs interact with multiple intracellular components, leading to the inhibition of essential cellular processes. These targets include cell wall biosynthesis, DNA and RNA synthesis, protein synthesis and folding, ribosomal function, enzymatic activity, and cell cycle regulation. Additional effects such as nucleic acid binding and degradation, mitochondrial dysfunction, and disruption of metabolic pathways contribute to the overall antimicrobial activity of AMPs.

Some AMPs can penetrate the cell membrane without causing lysis and can act on intracellular targets. Their intracellular action involves the inhibition of DNA, RNA and protein synthesis, as well as the promotion of the release of proteolytic enzymes and even the inhibition of metabolic enzymes (Figure 4) [112].

AMPs translocate to the membrane and bind directly to DNA and RNA, inhibiting the replication and transcription process [108]. The CF‐14 peptide has been shown to inhibit microbial growth by inhibiting the enzyme DNA gyrase, which is directly involved in DNA replication [112]. Another well‐described peptide is buforin II, which can penetrate lipid vesicles without affecting membrane permeability, acting directly on the histone H2A protein, which interacts with nucleic acids [113, 114].

Another intracellular mechanism that has been observed is the inhibition of protein synthesis, where AMPs interact with bacterial ribosomes or even auxiliary proteins and inhibit the translation of essential proteins [115]. Moreover, AMPs have been shown to inhibit translation initiation, impede the transition from the initiation complex to the DNA elongation phase, and hinder the protein folding step [116].

The PR‐39 peptide, an AMP rich in proline and arginine, can penetrate the outer membrane and, once inside, interrupt protein synthesis and trigger a process of protein degradation that is fundamental to the process of DNA synthesis [117, 118]. Another peptide derived from the apidaecin family and rich in proline is able to prevent the formation of 50S ribosome subunits, whereas another oncocin‐type peptide can limit mRNA translation by binding to the 70S ribosome. These affinities for specific ribosomes directly affect protein synthesis [116, 119]. In addition to inhibiting protein synthesis, AMPs can inhibit cytoplasmic enzymes that are essential for bacterial metabolism [92]. This inhibition directly interferes with the production of cell wall components in various microorganisms. This process involves the binding of peptides to lipid II, which results in the inhibition of the polymerization of cell wall peptidoglycan. Consequently, this leads to the failure of wall formation and, ultimately, cell lysis [120].

The post translationally modified microcin peptide J25, produced by ribosomes, has been shown to bind to the RNA polymerase secondary channel, inhibiting folding, which is essential for RNA polymerase catalysis [121]. Another peptide, LL‐37, has been demonstrated to inhibit the palmitoyltransferase PagP, which is responsible for restoring membrane permeability through lipid acylation [122]. Teixobactin has the capacity to bind to lipids II and III, which are precursors of the cell wall teichoic acid), inhibiting the synthesis of the cell wall of microorganisms [120]. The NP‐6 peptide, derived from Sichuan pepper seeds, has been demonstrated to inhibit metabolic enzymes, such as β‐galactosidase activity, affecting the ability to metabolize sugars [123].

### 3.3 Limitations and Challenges

A major challenge in developing AMPs as therapeutic agents lies in their vulnerability to proteolytic degradation, which significantly limits their stability and efficacy in biological systems. This degradation may occur through multiple pathways and is often determined by the peptide's own structural characteristics, including the presence of amino acid residues that increase susceptibility. Both endogenous proteases, such as those present in the cytosol of human erythrocytes, and bacterial proteases, including aureolysin, a metalloprotease capable of degrading certain AMPs, can contribute to this instability, ultimately impacting their therapeutic potential [124]. Moreover, the oral delivery of AMPs can be challenging owing to the potential for proteolytic degradation by enzymes present in the digestive tract, such as trypsin and pepsin. Additionally, systemic administration can result in a short half‐life in vivo due to degradation by proteases and hemolytic activity [125]. Another factor that limits the use of some AMPs is their cellular toxicity, which results from their interaction with cell membranes and consequent cell lysis [126].

### 3.4 Recent Advances

AMPs have a broad spectrum of activity and are being studied as alternative treatments to antibiotics and even in combination with other drugs. The gram‐positive bacterium *Mtb* is among the resistant bacteria being studied for treatment with natural and synthetic AMPs due to the increasing number of cases of resistance to conventional antibiotics in recent years [95, 127]. In the last five years, several studies have been performed with different AMPs showing activity against *Mtb*, and some of the results obtained are described in this review. In addition, some peptides with anti‐TB activity that have been studied over the years are described in Table 2 for comparative purposes.

**TABLE 2:** AMPs investigated within the last five years for their activity against *Mycobacterium* species, including sequence, antimicrobial activity, key highlights, and reference.

Peptide	Sequence	Activity	Highlights	References
		(MIC)		
**B1CTcu5**	LIAGLAANFLPQILCKIARKC	12.5 µg/mL (*Mtb*)	Penetrates host cells without exhibiting toxicity.	[128]
**Gran1**	QRSVSNAATRVCRTGRSRWRDVCRNFMRR	Reduced *Mtb* by 38% at 1–10 µM	Active against *M. kansasii, M. avium, and M. bovis*. Directly interacts with the mycobacterial cell wall. No toxicity observed in vivo or in vitro. Does not induce an inflammatory response.	[129]
**aRP557**	KCKKFCIGKYCVKWCFR‐NH2	64 µg/mL (*M. abscessus*)	Increases antibiotic sensitivity of *M. abscessus* growing in biofilms.	[130]
**D‐hLF 1‐11**	GRRRRSVQWCA	100 µg/mL (*Mtb* H_37_Ra, H_37_Rv); 200 µg/mL (INH‐ and RIF‐monoresistant, MDR *Mtb*)	Shows synergistic effects with RIF or INH, improving MIC values. Less than 1% hemolysis observed even at 4000 µg/mL.	[131]
**Tpl**	KWCFRVCYRGICYRRCRGK‐NH_2_	20 µm (*M. smegmatis*); 1.25 µm *(M. fortuitum, M. wolinskyi*)	Inhibits 90% of biofilm formation (40 µm) and achieves ∼85% eradication. 100% cell viability at 40 µm.	[132]
**LoP‐1**	CRWRWKCCKK‐NH_2_	10–20 µm (*M. smegmatis*); 20 µm (*M. wolinskyi*)	Inhibits 97% of biofilm formation (40 µm) and achieves 85% eradication. No cytotoxicity observed (40 µm).	[132]
**W3R6**	VWRRWRRFWRR‐NH_2_	12.5 µm (*Mtb* H_37_Ra)	Induces low levels of drug resistance in *M. smegmatis* (2‐fold after 10 passages). Low toxicity to macrophages. Inhibits TNF‐α and IL‐6 production.	[133]
**NZ2114**	GFGCNGPWNEDDLRCHNHCKSIKGYKKKYCAKGGFVCKC	6.1 µm (MIC_90_ for *M. bovis* BCG); 75 µm (MIC_90_ for *M. abscessus*)	No toxic effects on macrophages (25 µm). Maintains antimycobacterial activity after 3 h in serum. Exhibits synergy with INH or EMB.	[134]

Abbreviation: Minimum inhibitory concentration (MIC).

Following an evaluation of the significant contribution of macrophages to the development of alternative mechanisms for *Mtb* elimination, mainly through autophagy, Coyotl et al. [135] investigated the therapeutic potential of synthetic AMP IP‐1 (KFLNRFWHWLQLKPGQPMY) against *Mtb*, including MDR strains. IP‐1 exhibited a dual mechanism of action: it induced autophagy in mammalian cells at 10 µm and triggered TNF‐α secretion in macrophages while directly exhibiting bactericidal activity against *MTB*. At higher concentrations (50 µM), IP‐1 caused cell death, but 10 µm was nontoxic and sufficient to activate autophagy. In vitro, IP‐1 displayed strong antimicrobial activity, with minimum inhibitory concentrations (MICs) ranging from 20 to 48 µg/mL for the drug‐sensitive H_37_Rv strain and 10 to 80 µg/mL for the MDR clinical isolate CIBIN99. In infected macrophages, treatment with 8–32 µg of IP‐1 for five days resulted in a significant reduction in intracellular *Mtb*, as confirmed by CFU counting. In a progressive pulmonary TB mouse model, BALB/c mice infected with H_37_Rv or MDR CIBIN99 were treated intratracheally with 8 µg of IP‐1 every other day for one month. This regimen led to a marked reduction in the lung bacillary load and tissue damage, as measured by CFU enumeration and histopathological analysis. Moreover, IP‐1 treatment restored the expression of protective cytokines (TNF‐α and IFN‐γ) in the lungs. Mechanistically, IP‐1 was shown to bind ATP (affinity constant: 440 µm), reducing intracellular ATP levels, which in turn activated autophagy without disrupting mitochondrial respiration at effective concentrations. These findings highlight IP‐1 as a promising candidate for adjunctive TB therapy, particularly for MDR cases, owing to its combined bactericidal, immunomodulatory, and autophagy‐inducing effects.

Navolotskaya et al. [136] investigated the antitubercular activity of the synthetic peptide LKEKK, derived from sequences of human thymosin‐α1 and interferon‐α2, in a mouse model of TB induced by *Mycobacterium bovis‐bovinus* 8. The peptide was administered intraperitoneally at doses of 0.1, 1.0, and 10 µg/kg for five consecutive days, starting on day 20 postinfection. Compared with no treatment (3.8 ± 0.4) or INH treatment (2.6 ± 0.2), treatment with LKEKK significantly reduced the lung injury index (2.2 ± 0.3, 1.8 ± 0.2, and 1.4 ± 0.3 for each dose, respectively). Immunologically, LKEKK treatment restored the production of IL‐2 and IFN‐γ in splenocytes to levels similar to those of healthy animals while also reducing IL‐4 levels. The phagocytic activity of peritoneal macrophages increased markedly, from 4.6% in untreated infected mice to 29.8% and 38.5% in those treated with 1 and 10 µg/kg LKEKK, respectively—surpassing even the effect of INH (19.4%). Binding studies revealed that LKEKK maintained high affinity for the membranes of infected macrophages and splenocytes (Kd values of 18.6 and 16.7 nM, respectively). These results indicate that LKEKK not only reduces lung damage in experimental TB but also restores key immunological parameters and enhances phagocytic function, supporting its potential as a promising candidate for adjunctive therapy in TB, including drug‐resistant forms.

**FIGURE 5:**
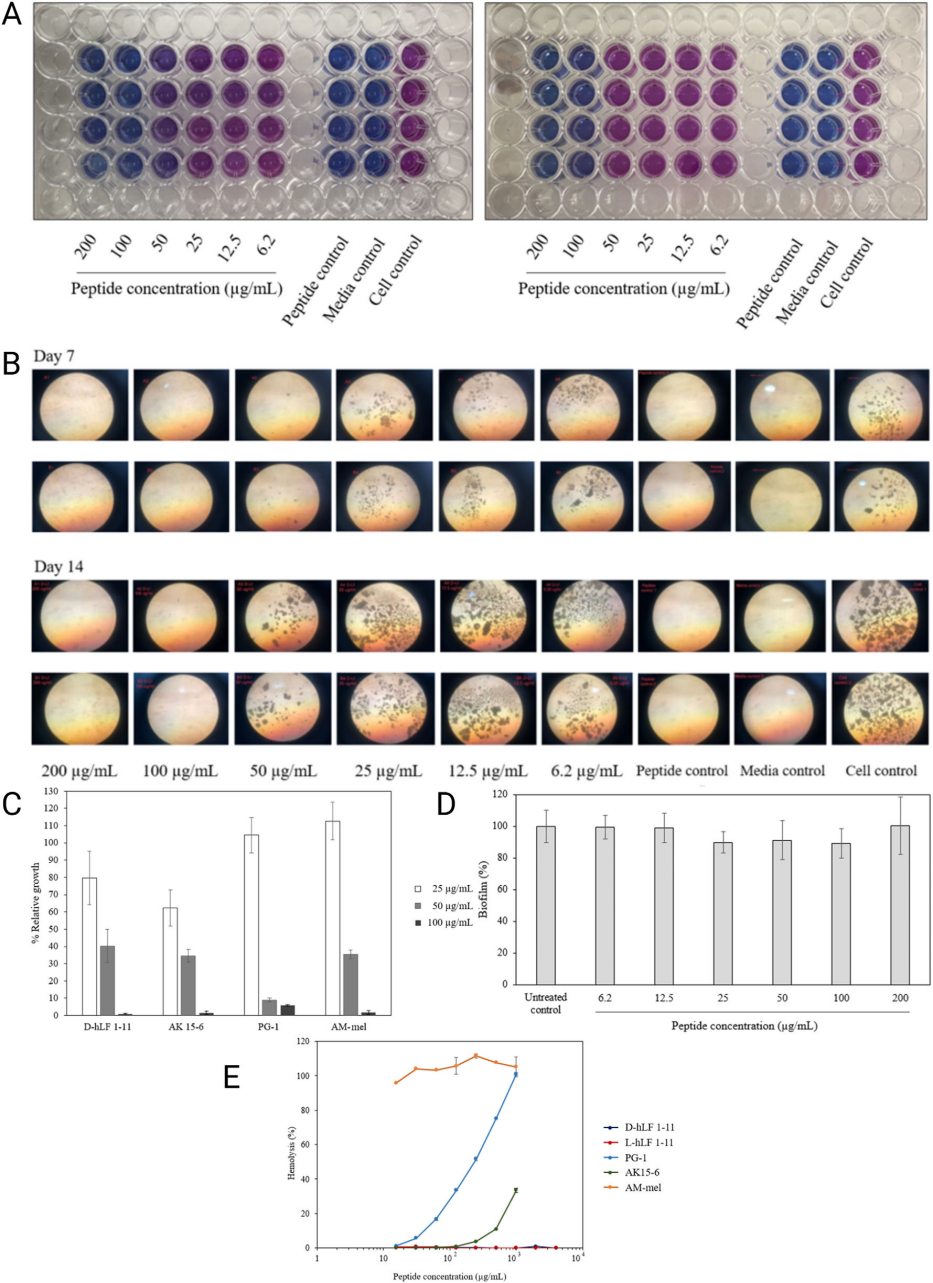
(A) The inhibitory effects of D‐hLF 1‐11 on multidrug‐resistant *Mtb* (left panel) and *M. avium* (right panel) were assessed via the REMA assay. Bacteria were treated with increasing concentrations of D‐hLF (6.2–200 µg/mL), and growth inhibition was determined by the absence of a color change from blue to pink, identifying the MIC as the lowest concentration with no color shift. (B) The MODS assay was employed to evaluate the activity of D‐hLF 1‐11 against *Mtb* H_37_Rv. Following incubation with the peptide at concentrations ranging from 6.2 to 200 µg/mL, bacterial growth was examined under an inverted microscope. The MIC was established as the minimal peptide concentration that fully suppressed cording or pellicle formation. (C) The efficacy of several AMPs against *Mtb* H_37_Rv was also measured via the MTT assay. Peptide concentrations of 25, 50, and 100 µg/mL were tested, and the results are shown as percentage growth relative to untreated controls. The data represent the means and standard deviations from three independent experiments. (D) The ability of D‐hLF 1‐11 to inhibit biofilm formation by *M. abscessus* was investigated via crystal violet staining. Preformed biofilms (4 days old) were exposed to peptide concentrations between 6.2 and 200 µg/mL. No statistically significant differences were found between treated samples and controls. The results are expressed as the means ± SDs of triplicate assays. (E) Hemolytic activity was assessed for various AMPs to determine their cytotoxicity toward human erythrocytes. The percent hemolysis was plotted against the logarithm of peptide concentration. The tested concentrations ranged from 15.6 to 1000 µg/mL for PG‐1, AK 15‐6, and AM‐mel and from 4000 µg/mL for both the L‐ and D‐forms of hLF 1‐11. Reprinted from an open‐access article by the MDPI [131]. Copyright 2022.

Intorasoot et al. [131] evaluated the antimycobacterial activity of the peptide D‐hLF 1‐11, derived from human lactoferricin, against both drug‐sensitive and drug‐resistant *Mtb* via several in vitro assays, including the resazurin microplate assay (REMA), the microscopic observation drug susceptibility (MODS) assay, and the 3‐(4,5‐dimethylthiazol‐2‐yl)‐2,5‐diphenyltetrazolium bromide (MTT) colorimetric assay. In REMA, D‐hLF 1‐11 completely inhibited bacterial growth in all *Mtb* strains (including multidrug‐resistant, INH‐monoresistant, and RIF‐monoresistant strains), as did nontuberculous mycobacteria, with an MIC of 100 µg/mL or higher. This inhibition was evidenced by the absence of a color change in the resazurin indicator at concentrations of 100 µg/mL and above (Figure 5A). The MODS assay further corroborated these results, establishing the MIC for D‐hLF 1‐11 at 100 µg/mL for drug‐sensitive strains and 200 µg/mL for MDR and monoresistant isolates. In this assay, the characteristic morphology of *Mtb* disappeared at inhibitory concentrations, confirming that bacterial growth was inhibited under microscopic observation (Figure 5B). The MTT colorimetric assay revealed that D‐hLF 1‐11 inhibited bacterial growth in a concentration‐dependent manner, with MICs of 100 µg/mL for susceptible strains (H_37_Rv and H_37_Ra) and 200 µg/mL for INH‐ and RIF‐monoresistant and MDR strains (Figure 5C). Notably, after 14 days of incubation, the D‐enantiomer of the peptide maintained its inhibitory activity, whereas the L‐form allowed for bacterial regrowth, indicating the superior stability of the D‐enantiomer. With respect to antibiofilm activity, *Mycobacterium abscessus* was used as a model organism. Crystal violet staining revealed no significant differences in biofilm biomass between groups treated with D‐hLF 1‐11 (ranging from 6.2 to 200 µg/mL) and untreated controls, indicating that the peptide did not display notable antibiofilm effects under the tested conditions (Figure 5D). Finally, hemolysis assays indicated that D‐hLF 1‐11 exhibited very low cytotoxicity, with less than 1% hemolysis observed even at concentrations as high as 4000 µg/mL (Figure 5E). This favorable safety profile, together with its in vitro antimycobacterial efficacy and high stability, position D‐hLF 1‐11 as a promising candidate for TB treatment, including multidrug‐resistant forms, although it does not show significant activity against mycobacterial biofilms.

## 4 Discovery of Amps via In Silico Tools

### 4.1 Computational Methodologies

Structural and dynamic studies are used to understand the functions of molecules, as they exhibit characteristics in each environment and over a predicted period [137]. Currently, computational approaches and deep learning are indispensable for drug discovery. Computational methodologies include contributions to the characterization of molecular mechanisms, ligand binding, identification of binding/activity sites, and refinement of structure—from binding positions to ligand‒target interactions [138].

In contrast to the traditional drug discovery approach, the understanding of the quantitative relationship between structure and biological activity, enabled by computational methodologies, has led to the emergence of computer‐aided drug design (CADD) applications. Moreover, more than 5,000 macromolecular structures attributed to *Mtb* have been deposited in the Protein Data Bank (PDB) via CADD strategies [139, 140]. These structures can be used in in silico studies for the rational design of drugs, as we will see below. Computational techniques can reduce drug production costs by up to 50%. To put it into perspective, bringing a drug to market costs, on average, between $500 and $800 million and taking 10‐15 years; however, the use of computational methods reduces these expenditures [139, 140].

Molecular docking is a computational tool that contributes to optimizing the discovery of new drugs and feeds databases on ligand interaction predictions. This structure‐based method is used in drug discovery. This silico tool allows the identification of new compounds with therapeutic potential, predicts ligand‒target interactions at the molecular level, and describes structure‒activity relationships (SAR) without the need to know the chemical structure of other modulators of the target [141]. For example, in the study by Mustafa et al. [142], the activity of AMPs was evaluated against different multidrug‐resistant bacterial strains with respect to the glucose‐1‐phosphate thymidyl transferase enzyme of the *Mtb* and H_37_Rv strains. Among the evaluated AMPs, the napin peptide showed the strongest interactions with the enzyme, with a binding score of ‐107.8 kcal/mol and twelve hydrogen bonds.

**FIGURE 6:**
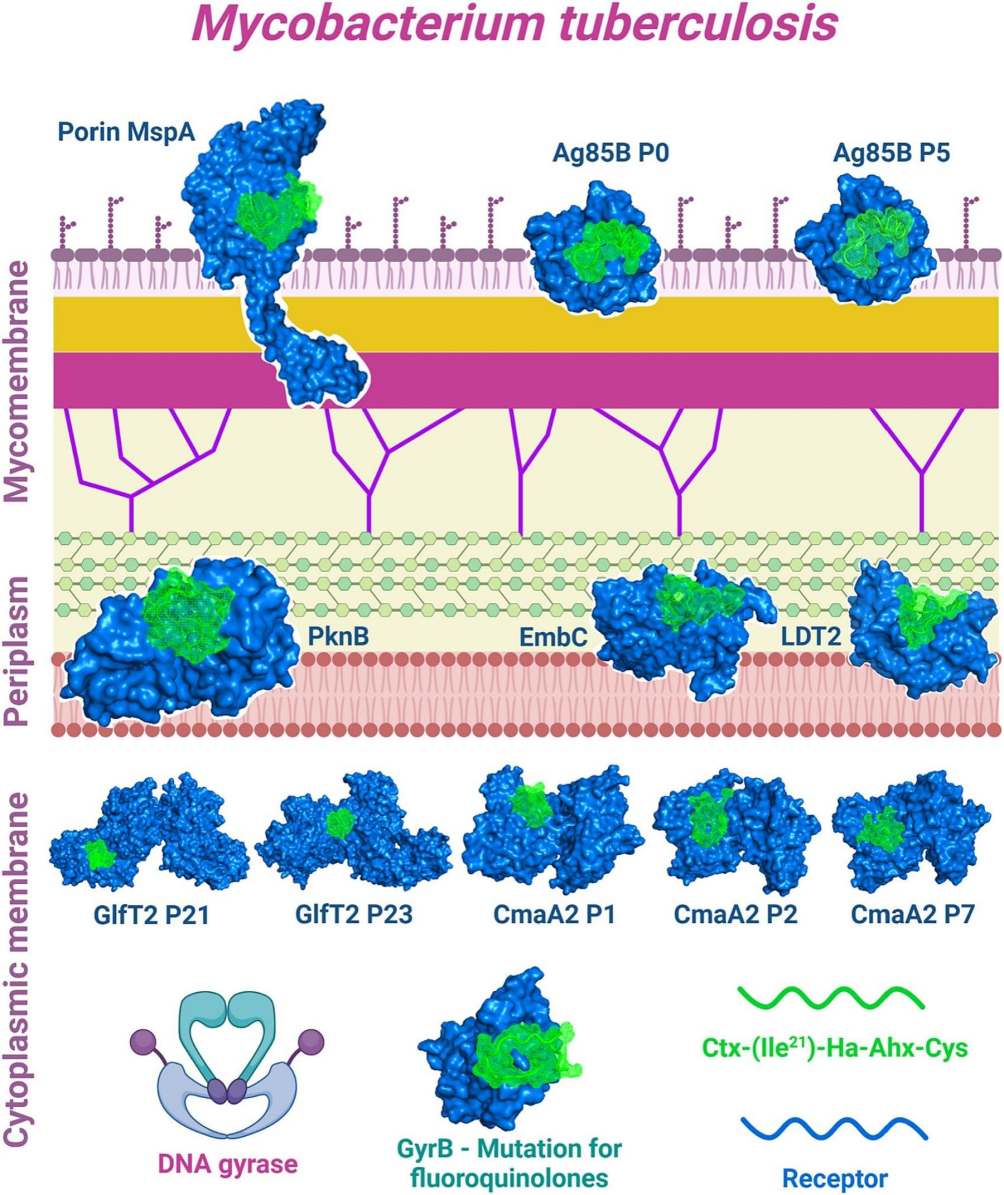
Molecular docking to assess the interaction between *Mtb* bacterial receptors (Ag85B, LTD2, GyrB, EmbC, GlfT2, Porin MspA, PknB, and CmaA2) is shown in blue, and the AMP Ctx(Ile^21^)‐Ha‐Ahx‐Cys (Ctx) is shown in green. Reproduced with permission. [143] Copyright 2024, Elsevier.

In the study by Primo et al. [143], the anti‐*MTB* activity of N‐acetylcysteine–chitosan‐based nanoparticles (NPs) loaded with RIF and conjugated with the AMP Ctx(Ile21)‐Ha was evaluated against clinical isolates (both multidrug‐resistant and extensively drug‐resistant) and the H_37_Rv strain. The modified chitosan and drug‐loaded NPs were characterized in terms of their physicochemical stability and antimycobacterial profile, showing potent inhibition with MIC values below 0.977 µg/mL. To investigate the potential mechanisms of action, an in silico study was conducted to assess the interaction of the peptide with *Mtb* membrane receptors. The molecular docking results revealed a total of 615 interactions between the ligand and the receptors. Simultaneously, 3D representations of the structures were generated via PyMOL (Figure 6). Using Discovery Studio Visualizer, various classifications, types, and quantities of interactions were identified.

As shown in Figure 6, the AMP Ctx(Ile^21^)‐Ha‐Ahx‐Cys (Ctx) exhibited interactions with key bacterial receptors. The docking study with the Ag85 receptor protein revealed three druggable pockets, with Pockets 0 and 5 being particularly noteworthy. Pocket 0 exhibited nine interactions with the ligand, including three hydrogen bonds and one π‐anion electrostatic interaction, with a remarkable simultaneous interaction between Asn170 and the ligand residues Phe14 and Ala15. Pocket 5 showed an even greater number of interactions (11 in total), with Lys11 of the ligand standing out by forming multiple connections with Phe232 and Ser235. The docking results for LDT2 and GyrB were less prominent, showing only five and seven interactions, respectively, and were mostly hydrophobic and of weak intensity. Finally, the docking study identified relevant interactions between the peptide ligand and various *Mtb* receptors, with particular emphasis on the Ag85B and CmaA2 proteins. In the Ag85B receptor, Pockets 0 and 5 exhibited the most significant interactions, including stable hydrogen and electrostatic bonds, especially those involving residues Asn170, Phe14, Ala15, Lys11, Phe232, and Ser235, suggesting strong affinity and inhibitory potential. In the CmaA2 receptor, two pockets established numerous interactions, including several hydrogen bonds (with Ser96, His141, Arg146, Ser135, Tyr147, and Gly72) and hydrophobic contacts, indicating a good fit of the ligand. Overall, the bioinformatics data suggest that the peptide has high potential to inhibit, primarily, the Ag85B protein and interact with other structurally relevant bacterial targets [143].

**FIGURE 7:**
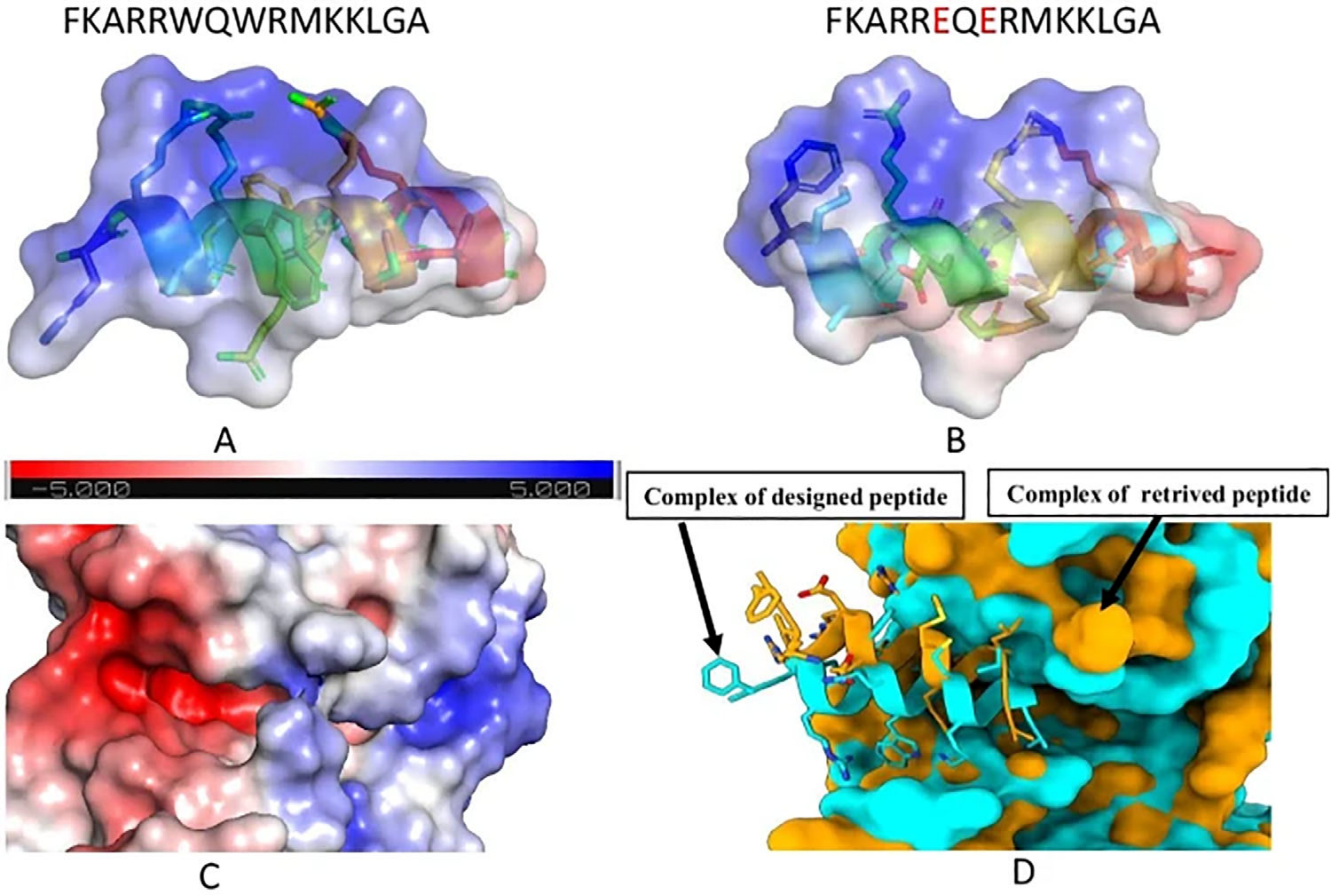
Modification of the SEQ578 peptide sequence. (A) Original peptide S578 of 15 amino acids. (B) The modified peptide at positions 6 and 8. (C) The pocket forms the cleft region of the protein. (D) Superimposition of the wild‐type complex and the substituted complex. Reproduced with permission. [144] Copyright 2024, Springer Nature.

Deka et al. [144] screened peptides with inhibitory activity against PknB—a serine/threonine protein kinase from Mtb—from the APD3 AMP Database, focusing on sequences of 5–25 residues. The target protein structure was visualized via PyMol and UCSF Chimera, and molecular docking was performed with the Autodock CrankPep Suite. Regardless of peptide length, certain residues—such as Glu15, Tyr94, and Asp102—consistently formed hydrogen bonds with the peptides, while longer peptides also engaged in salt bridges involving Asp96, Asp102, and Arg101. The peptide SEQ578 showed the highest binding affinity. Substituting tryptophan for glutamate at positions 6 and 8 altered the binding pocket topology (Figure 7), increasing the binding free energy from –7.76 to –13.65 kcal/mol. Figure 7A–D visualize the charge distribution and docking orientation, while interaction analysis highlighted hydrophobic contacts with Ile16, Gly18, Phe19, Val98, and Ile103 and hydrogen bonds and salt bridges with additional key residues. The selected docked complexes were further evaluated via MD simulations to assess their conformational stability and identify critical residues for peptide inhibition of PknB.

MD simulations have made significant advances in several scientific fields, especially in rational drug design. This computational method has proven effective in providing detailed characterization of biomolecular systems, complementing experimental data, optimizing designs, and predicting relevant properties for chemical systems. With respect to drug development, MD can be applied in simulations to characterize membrane structure and organization, membrane permeability, lipid‒protein interactions, lipid‒drug interactions, protein‒ligand interactions, and protein structure and dynamics [145]. Currently, drug discovery is enhanced by MD techniques, owing to efficient and accurate algorithms, greater ease of use, and the creation of large‐scale macromolecule structure databases. This computational tool enables efficient, cost‐effective molecular screening, reducing the resources spent on large‐scale phenotypic screening experiments, for example [146, 147].

**FIGURE 8:**
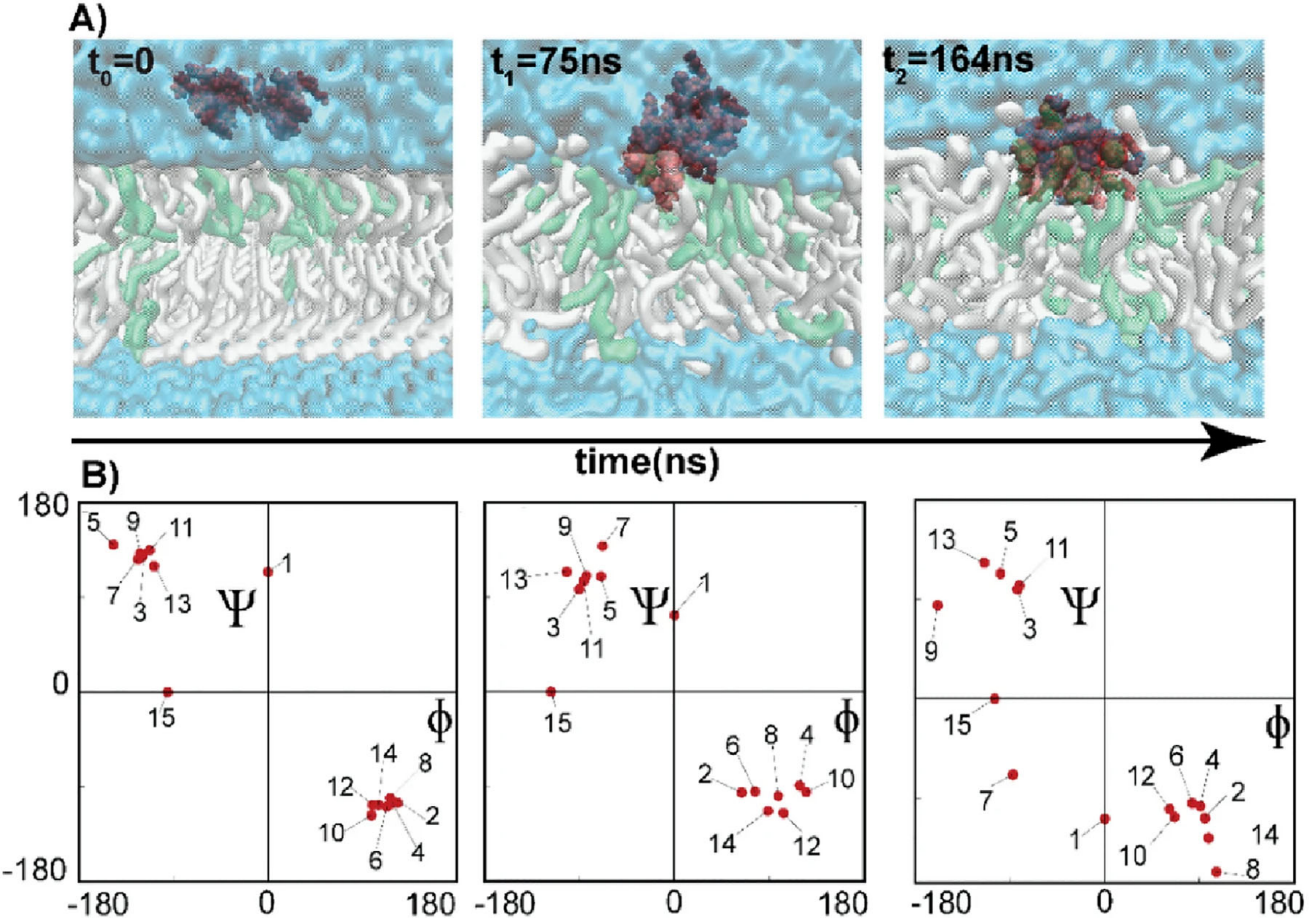
Interaction of AMP AS08 with the POPC: POPG membrane (3:1). (A) Insertion of peptide AS08 over time. (B) Ramachandran plot for the peptide at different time points (t0, t1, and t2). Reproduced with permission. [148] Copyright 2021, Springer Nature.

Hazam et al. [148] elucidated the molecular mechanisms by which AMPs interact with and disrupt mycobacterial membranes, ultimately supporting the rational design of new therapeutic agents against Mycobacterium species. To this end, the authors synthesized several AMPs (AS01–AS04, AS06, and AS08) via solid‐phase synthesis on a Rink amide resin and the Fmoc strategy. Following in vitro screening, AS03, AS05, AS06, and AS08 were identified as active against M. smegmatis—a nonpathogenic model for Mtb—and were subsequently selected for in silico simulations. Focusing on AS08, MD simulations were performed with a model membrane composed of POPC and POPG phospholipids to mimic the mycobacterial envelope. The results revealed a stepwise interaction: the peptide started in the aqueous phase (t_0_), adsorbed onto the membrane surface (t_1_), and ultimately penetrated the upper membrane layer (t_2_) (Figure 8A). Ramachandran plots at each time point (Figure 8B) illustrate the conformational transitions during membrane insertion. It was inferred that the driving force for peptide penetration was electrostatic attraction between the positively charged residues of the peptide, especially lysine, and the phosphate groups of the membrane's outer layer. Interestingly, peptides with lower MICs and greater antimicrobial potency, such as AS08 and AS03, tend to remain closer to the outer membrane surface. These results suggest that peptides with greater electrostatic potential exhibit stronger adhesion to the membrane, leading to enhanced lytic activity against mycobacterial cells.

In silico studies, such as docking and MD, have been applied to the rational design of new drugs. As previously described, these studies generally focus on the identification of molecules and their design, such as proteins or enzymes, which are crucial for the survival of infectious agents [149]. Considering target‐directed approaches, a group of molecules currently employed in these in silico studies are AMPs (AMPs). These molecules are regarded as promising candidates for novel antimicrobial therapies, especially against resistant strains, owing to their multiple mechanisms of action—which, rather than targeting a single molecule or metabolic pathway, make it difficult for bacteria to develop resistance to AMPs [150].

A recent example is the work by Hirano et al. [151], which described a rationally designed amphipathic helical peptide, named Stripe, focused on the distribution of natural cationic and hydrophobic amino acid residues. The authors investigated a set of foldamers based on this peptide, incorporating α,α‐disubstituted amino acids or side‐chain cross‐linking to stabilize the helical structures. The results highlighted a peptide containing 2‐aminoisobutyric acid (Aib) residues as a potent antimicrobial. Electrophysiological measurements demonstrated that this peptide could form stable pores in a 1,2‐dioleoyl‐sn‐glycero‐3‐phosphoethanolamine (DOPE)/1,2‐dioleoyl‐sn‐glycero‐3‐phosphoglycerol (DOPG) bilayer but not in a dioleoylphosphocholine (DOPC) bilayer.

The combination of advanced in silico techniques, such as those mentioned above, which are used for the rational design of drugs, consolidates as a tool for the development of new drugs. Today, artificial intelligence (AI) technologies and machine learning (ML) also play a role in discovering potential molecules and in drug development [152]. ML and deep learning (DL) algorithms have been implemented in several processes, including peptide synthesis, structure‐based virtual screening, ligand‐based virtual screening, toxicity prediction, drug monitoring and release, pharmacophore modeling, quantitative structure‒activity relationships (QSAR), drug repurposing, polypharmacology, and physicochemical activity [153].

Machine learning is a technology within AI that offers economical algorithms, reducing the time required to predict antimicrobial molecules. This technology has made significant advancements in areas such as AMP design and nanotechnology for delivery [154]. Another application is the use of evolutionary algorithms that simulate the evolution of sequences to generate peptide libraries oriented to diversity and optimize candidate peptides. Furthermore, proteogenomic analyses have been incorporated into computational workflows to discover AMPs from natural sources [155]. Machine learning can be divided into supervised and unsupervised approaches, depending on the use of labeled data. In supervised learning, the algorithm learns to distinguish between AMPs and non‐AMPs through repeated exposure, associating features such as charge and amphiphilicity with classification labels (antimicrobial or nonantimicrobial). In contrast, unsupervised learning involves analyzing unlabeled data to identify patterns that differentiate peptide classes [154].

In the absence of experimental data, AMP prediction models are employed to identify novel sequences, predict mechanisms of action or targets, and estimate 3D structures. These models mine peptide databases and the scientific literature to recognize features shared among known AMPs [155]. Peptide databases, in turn, provide physicochemical characteristics, modifications, mechanisms, and structural information, supporting both peptide design and the optimization of existing AMPs. Optimization efforts often focus on the physicochemical parameters involved in microbial membrane disruption—as seen in alpha‐helical peptides, whose amphipathic structure and net charge directly influence membrane permeabilization [154].

Aguilera‐Puga & Plisson [156] evaluated the mechanisms of action and the structural landscape of AMPs that destabilize membranes, peptides that penetrate membranes, and those that bind to proteins. By analyzing critical features such as dipeptides and physicochemical descriptors, the authors developed models with accuracies ranging from 86‐88% for these categories. This approach allowed for the prediction of peptide structures that would likely fold into α‐helices, spirals, or mixed structures—key structural aspects associated with the antimicrobial activity of these peptides.

Hassam et al. [157] used both active and inactive drug candidates to train machine learning models against pantothenate synthetase (PS), a potential drug target for Mtb. An artificial neural network was trained via the grid search method to optimize the hyperparameters. The authors calculated 1,444 molecular descriptors, but initially, twenty descriptors were selected on the basis of their significant Pearson correlation with the ‐log IC_50_ values. Six descriptors were identified as the most significant based on the r2 value of the multiple linear regression, namely, ATSC6m, ATSC6v, AATSC2m, MATS4e, n5 Ring, and nT5 Ring. The prediction of active molecules and their ‐log IC50 values showed that these models were stable enough to generalize and be used in future screening of compound libraries against PS, for example.

### 4.2 Databases and Virtual Screening

Structure‐based virtual screening offers a practical route for accelerating antimicrobial discovery by prioritizing candidates that are most likely to engage a tuberculosis‐relevant target. In this context, virtual screening is commonly combined with molecular docking to estimate receptor‐ligand binding and to rapidly triage large libraries into a short list for follow‐up, thereby reducing experimental burden while preserving chemical and sequence diversity [138, 158]. The performance of these workflows depends strongly on the underlying ligand sources, and AMP repositories are particularly valuable because they consolidate bioactive sequences and, in some cases, developability‐related annotations that can be used as early filters [159].

Several widely used AMP repositories, however, are broad‐spectrum resources rather than TB‐dedicated databases. The Data Repository of Antimicrobial Peptides (DRAMP) is an open‐access, manually curated platform that aggregates general, patented, clinical, and specific antimicrobial peptide entries without being organized around a TB‐specific subset [160]. In its latest release (DRAMP 4.0), the repository reports 30 260 total entries and expands developability‐related metadata by adding a stability dataset (110 entries with experimentally determined serum/protease stability or half‐life information) and updating toxicity fields, including 2891 entries with hemolytic activity and 2674 entries with cytotoxicity supported by experimental validation [161]. Likewise, the Database of Antimicrobial Activity and Structure of Peptides (DBAASP) integrates peptide sequences and chemical modifications with structural information, bioactivity, and toxicity annotations (including cytotoxicity/hemolysis) across multiple target classes rather than focusing specifically on Mtb [162]. To complement these broad‐spectrum repositories, AntiTbPdb is a dedicated knowledgebase of experimentally verified anti‐TB/anti‐mycobacterial peptides. It contains 1010 entries extracted from research papers and patents and includes strain‐specific mycobacterial targets together with reported inhibition concentration and related annotations. To complement these broad repositories with TB‐focused evidence, AntiTbPdb curates experimentally verified anti‐TB/anti‐mycobacterial peptides (1010 entries) and provides an explicit breakdown by mycobacterial species, with most records reported against Mtb (550) and Mycobacterium smegmatis (202), alongside additional coverage for Mycobacterium avium (60) and Mycobacterium bovis (50) [163].

Against this background, peptides remain compelling screening candidates because their size and conformational flexibility make them well suited to engage protein surfaces that are difficult to modulate with small molecules alone. Peptides are generally small molecules, making them excellent candidates as inhibitors. Proteins with large, smooth surfaces are more easily targeted by the conformational flexibility of these molecules [164].

The discovery of bioactive peptides typically requires high‐throughput screening of large libraries and in silico approaches, as these methods are efficient and cost‐effective. In the study by Kumari & Subbarao [165], high‐throughput virtual screening was performed to identify potential multitarget drug candidates against TB, such as murA, murB, murC, murD, murE, murF, murG, and murI, which are involved in peptidoglycan biosynthesis. In this virtual screening, 56 400 compounds from the ChEMBL library (another manually curated database of bioactive molecules with drug‐like properties) were analyzed, and the top 10 were identified as potential drug candidates. The most promising complex, CHEMBL446262, was subjected to MD simulation to evaluate its stability, and the binding energies of the best complexes were calculated. These ligands demonstrated high affinity and inhibitory potential against Mtb, indicating that multitarget therapies may be more effective than single‐target treatments. Table 3 summarizes examples of peptides designed and computationally evaluated by docking or molecular simulation against Mycobacterium species.

**TABLE 3:** Peptides designed and computationally evaluated by docking or molecular simulation against *Mycobacterium* species.

Peptide	Designed from	Sequence of the best peptide	Program/Software/Website	Target species	Description	References
PK34	Mycobacteriophage D29	NR	AutoDock 4.2	*Mtb* strains H_37_Rv, H_37_Ra, and BCG	Molecular target: Trehalose‐6,6‐dimycolate. Best activity, H_37_Rv MIC 12.5 µg/mL. Other activities, MIC > 12.5 µg/mL	[166]
Peptide based on LAMA2	LAMA2	NR	AutoDock‐Vina and Gromacs	*M. leprae*, strain not mentioned	Molecular target: Phenolic glycolipid‐1. Activity: no in vitro assays	[167]
MazE‐mt9 (α4)	MazEF toxin‐antitoxin system	NR	PHENIX.refine and PyMOL	*Mtb*, strain not mentioned	Molecular target: MazF‐mt1 and MazF‐mt9 toxins. Activity: no in vitro assays	[168]
hBD consensus and hBD10	hBD	hBD10: RECRIGNGQCKNQCHENEIRIAYCIRPGTHCCLQQ	UniProt Knowledgebase, Swiss‐Prot database, CLUSTALW and BLASTp	*Mtb* strain H_37_Rv and MDR clinical isolate	Molecular target: no. hBD10: best activity, MDR, MIC 5.9 µm. Other activities, MIC > 5.9 µm	[169]
1 St series and 2 nd series	Human variant of GST‐theta	2 nd series: 2.1: YRAMLLRIARIRMRL 2.2: YWWMLLRIWRIWMRL 2.3: RWWMLLRIWRIWMRL	PEP‐FOLD3	*Mtb* strain H_37_Rv	Molecular target: no. Best activity, 2.1, 2.2 and 2.3, MIC 3,12 µm. Other activites, MIC > 3,12 µm	[170]
Ctx(Ile21)‐Ha, Ctx(Ile21)‐Ha‐Ahx‐Cys, and others	Brevinin‐1	Ctx, GWLNVAKKIGKAAAFNVAKNF‐Ahx‐C	AutodockVina, Discovery Studio Visualizer, Pymol	*Mtb*, strains H_37_Rv and clinical isolates CF 169 and CF11	Molecular target: Bacterial receptors: Ag85B, LTD2, GyrB, EmbC, GlfT2, Porin MspA, PknB, and CmaA2. Best activity, H_37_Rv, MIC 7.57 µg/mL. Other activities, MIC >7.5 µg/mL	[143]
AS01, AS02, and others	NR	AS02: RKRWLWLKRKWLW	Delphi, MATLAB, Mem‐Builder versão 2.0., GROMACS 5.0.4, VMD, PyMol, GridMAT‐MD	*M. smegmatis* strain ATCC607	Molecular target: Bacterial membrane composed of phospholipids, POPC and POPG. Best activity, AS02, MIC 3,13 µm. Other activities MIC >3.13 µM	[148]
P1, P2, and others	—	P6: TSHLH	V‐life MDS, AutoDock4.2	*Mtb*, strain H_37_Rv and clinical MDR isolate *M. smegmatis*, strain mc(2)155	Molecular target: SBLs. Best activity, P6+Amp/Amx, MIC 0.5 mg/L. Other activities, MIC>0.5 mg/L	[171]
Anti‐Rho	C‐terminal region of Psu, the capsid protein of bacteriophage P4	16: MHHHHHHTPAQGMIFSVKURTGITSVSVKAWLFWRMREDFQPDTD 33: MHHHHHHTPAQFIFFRVKVRNWHTSVSVKAWLFWRMREDFQPDTD	Iterative Threading ASSEmbly Refinement, GROMACS‐2018, CHARMM 36, PROCHECK Ramachandran plot, Modeller 9v20, ClusPro, KFC, HotRegion	*Mtb*, strain not mentioned *M. smegmatis*, strain not mentioned *M. bovis*, strain not mentioned	Molecular target: transcription terminator Rho Best activity: Inhibition of the ATPase activities of the Rho proteins of *Mtb*: hydrolyzed in the presence of 50 µM of peptides 16 and 33. Inhibition of in vitro growth in *M. smegmatis* and *M. bovis* transfectants expressing peptide 33	[172]
Napin, snakin‐1, and others	Isolated from the seed storage protein of *Brassica* species	Napin: QPQKCQREFQQEQHLRACQQWIRQQLAGSPF	HADDOCK v.2.4, PyMOL Molecular Graphics System, PDBsum, Desmond, Protein Preparation Wizard, System Builder	*Mtb* strain H_37_Rv	Molecular target: RmlA. Best activity: no in vitro assays	[142]
S578, S785 and others	—	S578: FKARREQERMKKLGA	PLIP, PyMol versão 2.3.4, UCSF Chimera versão 1.6.1, Autodock Crank Peptide (ADCP), Gromacs, LigPlot	*Mtb*, strain not mentioned	Molecular target: PknB. Best activity: no in vitro assays	[144]
AKVUAM‐1 e AKVUAM‐2	—	AKVUAM‐1: YYYYEEKWW AKVUAM‐2: YYRRYHHHQ	ClusPro, Schrödinger, SwissADME, BIOVIA Discovery Studio	*Mtb*, strains H_37_Rv, ATCC 35838 (resistant to RIF), ATCC 35820 (resistant to STR), ATCC 35837 (resistant to EMB), 5 MDR‐TB isolates with determined resistance profiles, and 5 isolates susceptible to all primary anti‐TB drugs (IST). *M. smegmatis*, strain not mentioned	Molecular target: Macrophage surface receptor CR‐1, Surfactant D protein and, outer membrane protein Cpn T Best activity, AKVUAM‐1, against IST‐16, MIC 128 µg/mL; AKVUAM‐2, against ATCC‐STR, MIC 128.0 µg/mL. Other activities, MIC>512 µg/mL	[173]
WBCATH	Buffalo cathelicidin, *Bubalus bubalis*	GLPWILLRWLFFRG	GUI CHARMM,	*Mtb*, strain H_37_Rv and clinical MDR isolate	Molecular target: Cell membrane. Best activity, MDR, reduction of CFU by 98% compared to the untreated control group. Other activities, the reduction of CFU is <98% for the H_37_Rv and MDR strains	[174]

Abbreviations: Not reported (NR); Palmitoyl‐oleoyl‐phosphatidylcholine (POPC); Class A beta‐lactamases (SBLs); 1‐palmitoyl‐2‐oleoyl‐sn‐glycerol‐3‐phosphoglycerol (POPG); Protein kinase B (PknB); Glc‐1‐P thymidyltransferase (RmlA); Alpha‐2 laminin subunit of Schwann cells (LAMA2); Human β‐defensins (hBD); Glutathione S‐transferase (GST).

In summary, the use of computational tools such as docking and MD, ML, DL, AI, and AMP repositories has shown great potential in drug discovery for neglected tropical diseases, such as TB. Approximately 10 million people have been ill with TB each year since 2000. Given these factors and the ineffectiveness and rapid resistance of pathogens to current treatments, finding new, effective, and safe drugs for TB remains a priority [175].

Virtual screening of large drug databases, combined with ML or DL, can accelerate the discovery and development of anti‐TB drugs and reduce costs in stages that consume significant financial resources and time for development. In the future, the quality of these models can be improved with the use of larger and more diverse datasets. Good learning requires high‐quality data and a high‐quality learning model [176, 177]. In a few years, drug research and development data for learning will increase in quantity and complexity, requiring powerful predictive models based on AI, such as DL; however, challenges remain, including the assumption of linearity in structure‒bioactivity relationships and the limited chemical diversity of previously described models, such as AMP repositories [176, 177].

### 4.3 Limitations and Caveats of MD Membrane Models in *Mtb*

The cell envelope of *Mtb* is a multilayered, highly adaptive system in which the plasma membrane is coupled to a peptidoglycan–arabinogalactan scaffold and an outer mycomembrane that is continuously remodeled in response to environmental and host‐derived stresses [178].

A defining feature of this envelope is its waxy lipid‐rich barrier, dominated by mycolic‐acid–associated structures and complex glycolipids, which creates physicochemical properties that differ fundamentally from canonical phospholipid bilayers [179]. Because envelope composition shifts with growth state and dormancy, lipid remodeling can measurably alter membrane dynamics and drug partitioning, directly impacting apparent susceptibility and tolerance [180]. Consistent with this functional importance, starvation‐induced antibiotic tolerance in *Mtb* depends on production of the outer membrane lipid phthiocerol dimycocerosate, underscoring that specific envelope lipids can drive phenotypes relevant to treatment outcomes [181].

These envelope‐specific features impose nontrivial constraints on MD simulations, because mycobacterial envelope assemblies require lipid chemistries, geometries, and packing regimes that are not captured by generic membrane representations [182]. Accordingly, recent multiscale modeling of mycobacterial plasma membranes built around mannosylated phosphatidylinositol lipids shows composition‐driven self‐organization that motivates using organism‐informed membrane models when interpreting peptide–membrane interactions [183]. When simplified binary bilayers, such as POPC and POPG are used, restricting the membrane to one or two lipids can substantially shift key biophysical properties relative to higher‐complexity bacterial mimics [184]. For this reason, conclusions drawn from POPC/POPG‐based MD readouts (e.g., insertion depth, disruption, or permeability surrogates) should be benchmarked against mycobacteria‐specific experimental assays, including metabolic‐tag‐based measurements of small‐molecule permeation across the mycomembrane in live micobacteria [185].

Even when computational models appear highly accurate, their reported performance can be overly optimistic if evaluation does not stress‐test true out‐of‐distribution generalizability and if models are inadvertently tuned to dataset idiosyncrasies rather than transferable determinants of activity [186]. Data leakage, through duplicated or near‐duplicated sequences, shared scaffolds across folds, or preprocessing decisions that peek at the test set, can substantially inflate apparent accuracy and mislead conclusions about real‐world utility [187]. Moreover, many predictors show pronounced error spikes around structure–activity discontinuities (activity cliffs), which limits their ability to extrapolate reliably to genuinely novel chemical space or peptide variants [188].

In AMP prediction specifically, heterogeneous datasets, inconsistent definitions of negatives, and non‐uniform benchmarking practices can yield inflated metrics that fail to translate to external validation settings [189]. Because label noise and sampling bias can materially alter learned structure–property signals, rigorous curation, harmonization, and uncertainty‐aware learning should be treated as foundational steps rather than optional refinements [190].

Generative approaches based on large language models can produce fluent but unsupported outputs, so explicit uncertainty estimation and safeguards against over‐trusting a single generation are essential [191]. A practical way to improve verifiability is to couple language‐model generation to external chemistry tools and structured execution, which increases traceability and reduces brittle reasoning errors [192].

Crucially, strong in silico potency or surrogate endpoints do not guarantee in vivo efficacy or a safe profile across absorption, distribution, metabolism, excretion, and toxicity, contributing to persistent late‐stage attrition [193]. Accordingly, computational models should be framed as hypothesis generators and prioritization engines whose credibility depends on prospective TB‐relevant assays and iterative updating with experimental feedback [194].

These considerations indicate that in silico optimization alone is insufficient to ensure effective tuberculosis peptide therapeutics. The same mycobacterial envelope features that complicate molecular dynamics interpretation also limit peptide exposure at infected macrophages and granulomatous lesions in vivo. Consequently, beyond computational prioritization, formulation strategies that enhance stability and biodistribution are required. This provides the rationale for the following section on the use of nanosystems for peptide delivery.

## 5 Use of Nanosystems for Peptide Delivery

TB lesions generate distinct and dynamic microenvironments that reshape antimicrobial exposure and response, so delivery becomes part of the therapeutic problem rather than a secondary detail [195]. Nanocarrier design can be used to navigate biological barriers and tune where payloads accumulate and when they are released, which is central when the intended site of action is intracellular and lesion structured [196]. Nanoscale co‐delivery can also maintain joint availability of combination partners and preserve synergistic activity at the site of infection [197].

### 5.1 Mechanistic Basis of Synergy between AMPs, Nanocarriers, and Antibiotics

In TB, synergy between AMP and antibiotics manifests as a functional gain that cannot be accounted for by the simple addition of individual effects, but rather arises from pharmacodynamic interactions that amplify the overall activity of the combination [198]. In AMP–antibiotic combinations, a common mechanistic route is envelope sensitization in which peptides transiently disrupt barrier function and increase intracellular accumulation of antibiotics that otherwise penetrate poorly [199]. This route is particularly relevant in tuberculosis because the *Mtb* envelope is remodeled across growth states, stress responses, and host environments, which shifts permeability and can alter combination outcomes [200]. Because the baseline envelope is an unusually low‐permeability barrier, modest gains in influx can translate into substantial increases in effective exposure at intracellular bacterial targets [201].

Limited porin availability together with the hydrophobic mycomembrane imposes strong diffusion constraints on many agents, so barrier perturbation provides a direct mechanistic basis for permeability‐driven synergy [202]. Permeability is not the only lever, since inducible efflux systems in *Mtb* contribute to intrinsic resistance phenotypes and influence how resistance can emerge under drug pressure [203]. In that context, verapamil and norverapamil have been shown to potentiate anti‐TB drugs and to counter efflux‐linked tolerance programs associated with macrophage stress [204].

Nanocarriers can reinforce these effects by co‐localizing payloads in infected cells and intracellular niches, raising local antibiotic and peptide availability where *Mtb* persists [205]. Macrophage‐targeted mesoporous silica nanoparticles illustrate this logic by trafficking into acidified endosomal compartments and delivering high local concentrations of antituberculosis drugs within infected macrophages [206]. Carrier composition can also matter in its own right, as β‐cyclodextrin nanoparticles have been reported to show intrinsic antibacterial activity while functioning as drug delivery platforms against *Mtb* [207]. Co‐encapsulation further supports combination performance by synchronizing the spatial and temporal availability of each component, which is difficult to maintain with separate formulations [197]. This synchrony is important in TB because antibiotic exposure is heterogeneous across lesions and microenvironments, so mismatched local concentrations can create windows of functional monotherapy that erode combination effects [208]. Host‐directed contributions can also contribute at the infected‐cell level, since cathelicidin‐associated autophagy pathways enhance intracellular killing and can complement antibiotic action in macrophages [209].

Carrier architecture dictates barrier transport, cellular uptake, and release kinetics, so TB nanomedicine studies commonly organize platforms by material class [210]. The next subsection follows that structure and moves from lipid‐based systems to polymeric, inorganic, and hybrid carriers explored for peptide delivery.

### 5.2 Types of Nanosystems Used

The development of nanosystems for drug delivery has expanded the therapeutic potential of AMPs, enabling more effective, targeted, and stable formulations [211]. The most used nanosystems for AMP delivery, lipid‐based, polymeric, and inorganic, share several advantageous characteristics, including the ability to improve peptide stability, protect against enzymatic degradation, and enhance cellular uptake [212]. These systems also enable controlled release and can be engineered for biocompatibility, making them suitable platforms for combating infections (Table 4). Despite these shared features, each class of nanosystem presents unique attributes that can be leveraged for specific therapeutic purposes. Lipid‐based nanosystems, for example, facilitate membrane fusion and enhance intracellular delivery, which is particularly important for targeting *Mtb* residing within host macrophages [213, 214]. Polymeric nanoparticles offer tunable drug release profiles and can be designed to display mucoadhesive or stimuli‐responsive behaviors, improving retention and bioavailability at mucosal sites [215, 216]. Inorganic nanoparticles possess distinctive physicochemical properties—such as magnetic, thermal, optical, and catalytic functionalities—that provide multifunctional capabilities and are increasingly explored in theranostic strategies for TB treatment [214]. By tailoring each carrier to the physicochemical properties of the peptide, such as charge, hydrophobicity, molecular size, stability, infection site, and desired release kinetics, it is possible to achieve more precise, stable, and effective antimicrobial intervention [217].

**TABLE 4:** Nanosystems fabricated in the last five years for AMP delivery against *Mycobacterium* species: physicochemical characteristics and biological efficacy.

Nanosystem	Size (nm)	Zeta potential (mV)	In vitro effect	Dose and animal model	In vivo effect	References
**HA@ LLKKK18**	133 nm	−23.5 ± 0.5	Reduced mycobacterial load in *M. avium* and *Mtb* infected macrophages; lowered IL‐6 and TNF‐α	Intratracheal, *Female C57BL/6* mice, every other day for 5 or 10 doses	Treatment significantly reduced lung bacterial burden (∼1.2‐log for *Mtb*, ∼1‐log for *M. avium*) after 5–10 alternate‐day administrations in mice, demonstrating effective pulmonary targeting and infection control	[253]
**AgNPs@NK‐2/AgNPs@LLKK‐18; NP‐1 and NP‐2**	NP1 (50 nm TEM), NP2 (100 nm TEM)	−2.70 and 13	AgNPs at 10 µg/mL reduced *M. smegmatis* CFU by 2 log_10_ and *M. marinum* by 1.5 log_10_ after 24 h. No significant cytotoxicity was observed in RAW 264.7 macrophages at concentrations up to 20 µg/mL, with cell viability > 90%.	NA	NA	[276]
**DMSN@NapFab**	163 nm, pore size 4.7‐10 nm	−17.5 ± 0.4	Enhanced intracellular uptake and killing of *Mtb* in macrophages	NA	NA	[274]
**MSN@NZX**	200 nm, pore size of 3 nm	−26	Fluorescently labeled MSNs (Atto488‐MSNs) showed rapid and efficient uptake by immune cells. At 300 µg/mL, >95% of primary macrophages and 80% of THP‐1 cells internalized particles within 30 minutes. At 25 µg/mL, uptake was 80% in primary macrophages and 40% in THP‐1 cells. Confocal microscopy confirmed dose‐dependent internalization after 2 h, more pronounced at 50 µg/mL than at 5 µg/mL. TEM revealed intact particles in vacuoles at early timepoints and intracellular degradation after 72 h. In MIC assays, MSN@NZX exhibited significantly stronger inhibition of intracellular *Mtb* in macrophages compared to the free NZX peptide	Female *BALB/c* mice model; lung infection with *Mtb* H_37_Rv via the intranasal route, 300 and 25 µg/mL of MSN	Treatment with MSN@NZX by intratracheal administration resulted in an 88% reduction in lung bacterial load compared to untreated controls (p = 0.0159). Mice treated with free NZX had an 84% reduction (p = 0.0079), and those treated with RIF showed a 90% reduction (p = 0.0079). The initial lung burden was 520 ± 32 CFU, which rose to 6.3 × 10^5^ CFU after 14 days in untreated animals. No significant difference was found among the NZX, MSN@NZX, and RIF‐treated groups (p = 0.0004 overall).	[275]
**Lp@Bcn5**	NA	NA	Lp@Bcn5 showed potent antimycobacterial activity, with a ≥3‐fold greater inhibition of intracellular *Mtb* in murine macrophages compared to control (p < 0.001). It was effective at a noncytotoxic concentration of 0.1 mg/L, which is also within the MIC_90_ range of the peptide. This effect was stronger than RIF at 1 mg/L. LDH assays confirmed no cytotoxicity at the active concentrations.	*C57BL/6JCit (B6)* mice infected with *Mtb strain* H_37_Rv, acute infection 10 mg/mouse of Bcn5 i.v. per day for 5 consecutive days starting day 1 postinfection.	Intravenous administration of Lp@Bcn5 (10 µg/mouse/day for 5 days) resulted in a mean survival time (MST) of 28 ± 3 days, representing a 7‐day increase compared to untreated mice (MST: 21 ± 3 days) (p < 0.05). This survival benefit was achieved at a 10‐fold lower dose than RIF (75 µg/mouse/day), which yielded MST of 34 ± 6 days.	[235]
**NP‐pRIF**	285 ± 11	−22 ± 0.5	NP‐pRIF significantly enhanced macrophage uptake, achieving up to 3.6‐fold higher internalization compared to nonfunctionalized NLCs. Both NP‐pRIF and NP‐RIF showed 2‐fold greater. antimycobacterial activity than free RIF, with MIC reduced from 1.0 to 0.48 µg/mL. The formulation was noncytotoxic at all tested concentrations and provided sustained RIF release (∼18% over 72 h), preserving over 80% macrophage viability.	NA	NA	[234]
**PLGANPs@B5**	206.6 ± 26.6	−27.1 ± 1.5	(PLGANPs@B5) significantly enhanced cytokine production in J774A.1 macrophages after 24 h, inducing higher TNF‐α and IL‐10 levels compared to free B5 or PBS‐NPs. IL‐1β production was similar between B5 and PLGANPs@B5 groups. These results indicate that PLGANPs@B5 induce a balanced pro‐ and anti‐inflammatory response, demonstrating immunostimulatory potential in vitro.	*female BALB/c* mice infected with *M bovis* challenge	Induced stronger immune response (↑TNF‐α, IgA, CD4^+^) and reduced lung/spleen bacterial load post‐*M. bovis* challenge; enhanced BCG protection, but free B5 peptide was more effective with RIF	[251]
**PNAP@MIAP**	312.6 ± 17.8 nm	−3.22 ± 0.048	PNAP@MIAP at 100 µM reduced intracellular *Mtb* (H_37_Rv) by ∼2.6 log_10_ CFU after 96 h in infected macrophages, compared to ∼1.3 log_10_ CFU reduction for free MIAP. The combination of PNAP@MIAP + INH (1 µg/mL) showed a 2.01‐fold greater reduction in CFU than PNAP@MIAP alone. PNAP@MIAP also modulated macrophage apoptosis and restored programmed cell death responses in infected cells without inducing necrosis.	NA	NA	[277]
**PCLNP@HHC‐8/PCLNP@MM‐10**	376.5 ± 14.9 (PCLNP@HHC‐8), 289.87 ± 17.98 (PCLNP@MM‐10)	26.20 ± 0.43 (PCLNP@ HHC‐8) and 15.82 ± 0.76 (PCLNP@MM‐10)	Decreased MIC against *M. smegmatis* and *Mtb*; synergism with RIF	NA	NA	[252]
**CSNPs@Pep‐H/AuNPs@Pep‐H**	CSNPs@Pep‐H: 244 nm; AuNPs@Pep‐H: 20 nm	+12	Free Pep‐H at 10 µg/mL showed ∼92% inhibition of extracellular *Mtb* (MIC = 10 µg/mL). In infected macrophages, Pep‐H at 5 µg/mL achieved a 1 log_10_ (91%) reduction in intracellular CFU. CSNPs@Pep‐H and AuNPs@Pep‐H significantly enhanced this effect: CSNPs@Pep‐H at 0.5 µg/mL reduced bacterial load by 80% vs. 12% for free peptide, and AuNPs@Pep‐H at 1 µg/mL achieved 91% reduction, compared to 45% for free Pep‐H. All formulations showed low cytotoxicity (<20%) and >80% cell viability up to 100 µg/mL. Nanoformulations also modulated host response by increasing IFN‐γ and RNOS, while reducing TNF‐α, IL‐6, and MCP‐1 levels.	NA	NA	[278]
**(Ctx‐CSNPs@RIF)**	123.9‐248.9 nm	+37	Potent inhibition of clinical *MTB* isolates, including XDR strains	NA	NA	[143]

Abbreviations: Not available (NA); hyaluronic acid nanogel loaded with LLKKK18 (HA@LLKKK18); biogenic silver nanoparticles loaded with NK‐2 (AgNPs@NK‐2); biogenic silver nanoparticles loaded with LLKK‐18 (AgNPs@LLKK‐18); AgNPs synthesized by A. macrophylla (NP‐1); AgNPs synthesized by Trichoderma sp. (NP‐2); dendritic mesoporous silica nanoparticles loaded with NapFab (DMSN@NapFab); mesoporous silica nanoparticles loaded with NZX (MSN@NapFab); bacteriocin complex (Bcn5); liposomes loaded with Bcn5 (Lp@Bcn5); tuftsin‐functionalized nanostructured lipid carriers loaded with rifampicin (NP‐pRIF); bovine neutrophil β‐defensin‐5 (B5); PLGA nanoparticles loaded with B5 (PLGANPs@B5); porous nanoparticle aggregate particles loaded with MIAP‐peptide (PNAP@MIAP); poly‐Ɛ‐caprolactone nanoparticles loaded with HHC‐8 (PCLNP@HHC‐8); poly‐Ɛ‐caprolactone nanoparticles loaded with MM‐10 (PCLNP@MM‐10); gold nanoparticles loaded with Pep‐H (AuNPs@Pep‐H); Chitosan nanoparticles loaded with Pep‐H (CSNPs@Pep‐H); Ctx(Ile21)‐Ha‐conjugated N‐acetylcysteine‐chitosan nanoparticles loaded with rifampicin (Ctx‐CSNPs@RIF).

Lipid‐based nanosystems include liposomes, solid lipid nanoparticles (SLNs), and nanostructured lipid carriers (NLCs), each presenting distinct structural and functional characteristics that influence loading capacity, release behavior and biological performance [218].

Liposomes, which are formed by one or more phospholipid bilayers surrounding an aqueous core, are highly versatile, as they can encapsulate both hydrophilic and lipophilic agents [219, 220]. The biomimetic nature of AMPs, which resemble the cell membrane, enhances their cellular uptake and fusion with host cell membranes, facilitating their intracellular delivery [221, 222]. Despite known limitations such as physical instability, including leakage and aggregation over time, these nanosystems remain among the most clinically useful. Their long‐standing safety record, scalable manufacturing, and regulatory familiarity contribute to their continued relevance [223].

SLNs, which are composed of a solid lipid core stabilized by surfactants, offer improved physical stability and controlled release profiles due to the crystalline matrix [224]. They are generally biocompatible and exhibit low cytotoxicity toward mammalian cells. Additionally, a solid lipid matrix provides protection against enzymatic degradation, improving the stability and bioavailability of encapsulated AMPs [225]. However, SLNs often suffer from limited drug loading capacity because of their highly ordered crystalline core, which restricts the accommodation of active compounds. This can also lead to drug expulsion during storage, posing challenges for long‐term formulation stability [226, 227, 228].

Compared with SLNs, NLCs, which are composed of a mixture of solid and liquid lipids, offer increased colloidal stability, increased drug loading capacity, and reduced crystallinity, which collectively improve long‐term stability [229]. Like SLN, the predominant lipophilic nature of the lipid matrix can limit the entrapment of polar peptides, resulting in low encapsulation efficiency. Like other systems can be functionalized with ligands such as lactoferrin, transferrin, or hyaluronic acid for active targeting of infected macrophages [230, 231]. Despite their advantages, challenges remain in achieving high encapsulation efficiency for certain AMPs and maintaining formulation stability under physiological conditions [220, 232, 233].

**FIGURE 9:**
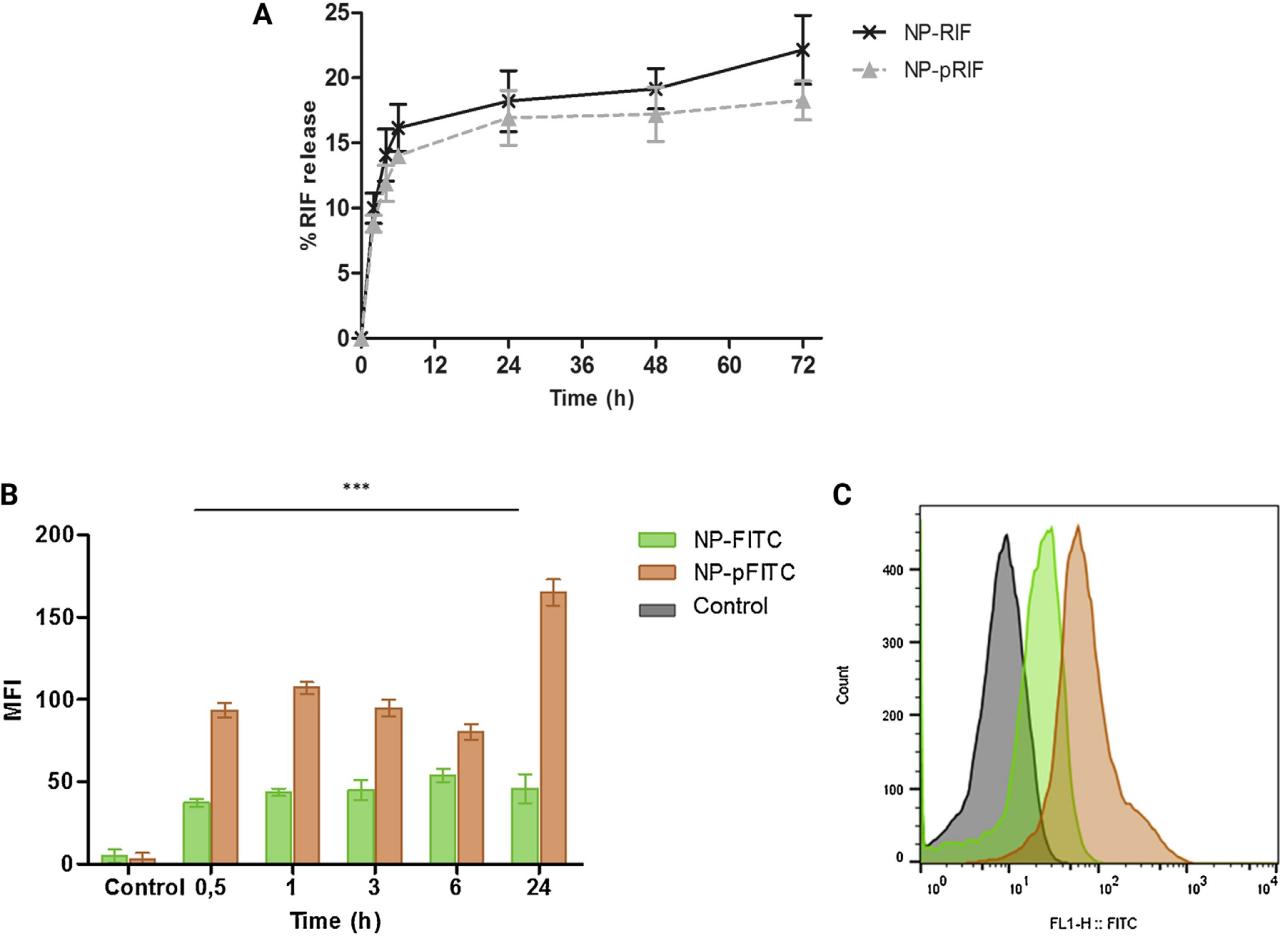
(A) Profile of the cumulative percentage of release of the NP‐RIF and NP‐pRIF formulations in PBS medium (pH 7.4). (B,C) Enhanced uptake of nanoparticles by macrophages, as determined by flow cytometry, demonstrating the mean fluorescence intensity over time and a fluorescence shift histogram. Reproduced with permission. [234] Copyright 2019, Elsevier.

Carneiro et al. [234] designed RIF‐loaded NLCs functionalized with a tuftsin peptide modified with an oleic acid molecule (pTUF‐OA) to improve targeting and intracellular delivery to alveolar macrophages. The peptide, composed of the sequence RPKT conjugated to oleic acid at the N‐terminus of arginine, was synthesized via Fmoc solid‐phase synthesis and subsequently incorporated into the lipid matrix during nanoparticle preparation, enabling its localization at the particle interface for effective receptor interaction. The NLCs were prepared via the microemulsion technique using 4% stearic acid and 1% oleic acid, 2.5% Phospholipon 80H and 2.5% Tween 80. RIF and pTUF‐OA (5 mg/mL each) were codissolved in the lipid phase prior to emulsification. The resulting formulation, referred to as NP‐pRIF, was obtained by dispersing the hot microemulsion into cold water. A control formulation without peptide (NP‐RIF) and other unloaded controls were also produced for comparison. NP‐RIF presented a diameter of 210 nm and a ZP of −31 mV, whereas NP‐pRI presented a diameter of 285 nm and a zeta potential (ZP) of –22 mV, reflecting the successful surface localization of the cationic peptide (rich in lysine and arginine residues). Both formulations were monodisperse (PDI < 0.2) and showed high encapsulation efficiency for RIF (NP‐pRIF: 81%) and a drug loading of 7.4 mg per 100 mg of lipid. Stability tests were conducted over 60 days at 4°C. No significant changes were observed in particle size, PDI, or zeta potential, indicating excellent colloidal stability. In vitro release assays revealed that NP‐pRIF had a biphasic release profile, with approximately 18% of the RIF released over 72 h, enabling sustained drug delivery (Figure 9A). Flow cytometry analysis revealed that, compared with nonfunctionalized NLCs, FITC‐labeled NP‐pRIF was internalized by macrophages 2.5‐fold more efficiently after 30 min of incubation, reaching 3.6‐fold greater internalization after 24 h (Figure 9B,C). Importantly, the nanosystems displayed low cytotoxicity and enhanced antimycobacterial activity, reducing the MIC values (0.48 µg/mL) against *Mtb* more effectively than free RIF (1.0 µg/mL). These findings underscore the potential of tuftsin‐functionalized NLCs to act as selective and potent delivery systems for TB therapy through macrophage targeting.

Sosunov et al. [235] investigated the antimycobacterial efficacy of class IIa bacteriocin (Bcn) cationic peptides naturally produced by gram‐positive bacteria. Five Bcns were isolated from bacterial culture supernatant and purified by gel filtration chromatography. In in vitro assays, among the five Bcn strains tested, four (Bcn2, 3, 4, and 5) demonstrated superior antimycobacterial effects compared with RIF, with ten‐fold reductions in the MIC_50_ and MIC_90_ values. The isolate Bcn5 was selected because of its potent antimycobacterial activity and low cytotoxicity to macrophages at 0.1 mg/L. Despite these robust inhibitory effects against extracellular *Mtb*, free Bcn5 exhibited no significant intracellular activity, which was attributed to its inability to cross macrophage membranes. To address this limitation, the peptide was incorporated into liposomes composed of phosphatidylcholine:cardiolipin (1:4 molar ratio) via the lipid film hydration method. Briefly, lipids were dissolved in ethanol, evaporated under vacuum, rehydrated in saline, and mixed with Bcn5 (10 mg/mL). After gentle stirring, the suspension was extruded (LiposoFast) to produce uniform liposomes. The resulting Bcn5‐loaded liposomes (Lip‐Bcn5) had an average diameter of ∼150 nm and encapsulated the peptide during bilayer formation (i.e., not via post synthesis surface functionalization). The Lip‐Bcn5 formulation showed good colloidal stability and did not induce LDH release (a marker of cytotoxicity) in infected macrophages. In vitro infection assays using peritoneal murine macrophages demonstrated that Lip‐Bcn5 reduced the intracellular replication of *Mtb* by at least three‐fold at 0.1 mg/L, outperforming free Bcn5 (which had no effect at this concentration) and RIF (tested at 1 mg/L). The control liposomes without peptide exhibited no antimycobacterial activity, confirming that their efficacy was due to the encapsulated Bcn5. In vivo, mice infected intravenously with 2 × 10^7^ CFU of *Mtb* were treated with Lip‐Bcn5 (10 mg/mouse/mouse for 5 days), and the treatment increased the mean survival time from 21 to 28 days, which was a statistically significant improvement (*p* < 0.05) and comparable to that of free RIF (75 mg/mouse, MST: 34 days). In contrast, free Bcn5 and empty liposomes did not prolong survival, suggesting that liposomal encapsulation was essential for therapeutic efficacy. These results demonstrate that AMP liposomal encapsulation enables their intracellular delivery and sustained antimycobacterial action, highlighting the importance of nanocarrier‐mediated strategies in AMP‐based TB treatment.

#### 5.2.1 Polymeric Nanoparticles

Polymeric nanoparticles are colloidal carriers made from natural or synthetic polymers, which may be biodegradable or nonbiodegradable materials, depending on their composition. Their structural versatility allows precise control over size, surface charge and degradation kinetics, making them particularly attractive for AMP delivery [236]. Among the most used polymers are cellulose, chitosan, alginate, and poly(lactic‐co‐glycolic acid) (PLGA) [237, 238].

Chitosan (CS) and alginate are natural polysaccharides widely investigated for AMP delivery because of their biocompatibility, low toxicity, and functional versatility. CS provides strong mucoadhesive properties and intrinsic antimicrobial activity, making it especially suitable for pulmonary or mucosal delivery routes [239]. Its positive surface charge facilitates electrostatic interactions with negatively charged bacterial membranes and host cell surfaces, promoting uptake and retention. However, batch variability and low solubility at physiological pH can limit its reproducibility and systemic application [240]. Alginate, an anionic polysaccharide obtained from brown algae, is frequently combined with chitosan to form polyelectrolyte complexes that improve mechanical stability, modulate release kinetics, and enhance biological performance. This combination allows for AMP encapsulation under mild, aqueous conditions, which is critical for preserving the structural integrity and bioactivity of sensitive peptides [241, 242].

PLGA is an FDA‐approved synthetic copolymer that offers excellent mechanical strength, controlled degradation kinetics, and tunable degradation rates. Its physicochemical properties can be adjusted through manipulation of its lactide:glycolide ratio and molecular weight, enabling customized release profiles [243]. Unlike natural polymers such as chitosan or alginate, PLGA offers superior structural stability and is particularly suitable for parenteral administration and systemic circulation [244]. However, PLGA‐based systems often exhibit initial burst release, especially when encapsulating hydrophilic peptides, and typically require organic solvents during fabrication. If not carefully optimized, these conditions can compromise the structural integrity of sensitive AMPs. Therefore, formulation strategies such as peptide hydrophobization, double emulsion techniques, or polymer blending are frequently employed to mitigate these drawbacks [245].

A critical distinction between these systems lies in the origin of the polymer, which affects its physicochemical behavior and therapeutic applicability. Natural polymers such as chitosan and alginate are highly biocompatible and ecofriendly, offering functional groups for mucoadhesion, immunomodulation, and pH sensitivity [246]. Nevertheless, they may suffer from batch‐to‐batch variability and limited mechanical strength. In contrast, synthetic polymers such as PLGA provide reproducibility, tunable degradation profiles, and greater control over release kinetics, although they often involve complex fabrication procedures and potential cytotoxicity from residual solvents [247]. Overall, polymeric nanoparticles offer a balance of structural versatility, functionalization potential, and biodegradability, enabling their application in host‐directed and sustained AMP‐based TB therapy [216, 248, 249, 250].

**FIGURE 10:**
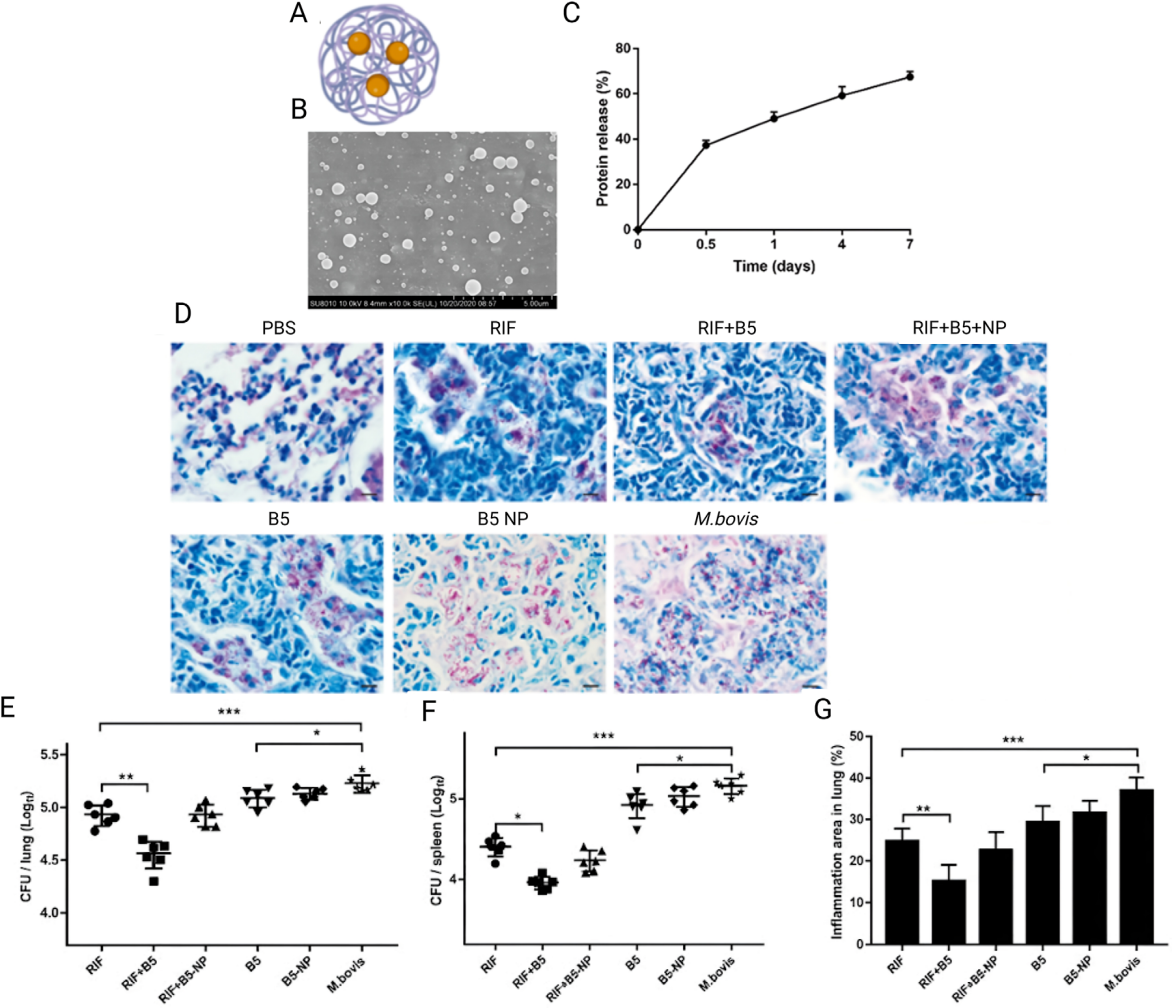
(A) Schematic representation of the formulated nanoparticle. (B) Scanning electron microscopy image showing the morphology of B5‐NPs. (C) In vitro release profile of B5 protein from B5‐NPs incubated in PBS (50 mg/mL) at 37°C for different time points, quantified by micro‐BCA assay. An initial burst release (>45% within 24 h, inset) was followed by sustained release, reaching ∼68% at day 7 (main graph). (D) Histopathological evaluation of lung tissues using acid‐fast staining, performed four weeks after *M. bovis* challenge. (E) Viable bacterial counts in lungs two weeks after treatment with B5 and/or rifampicin. (F) Viable bacterial counts in spleen two weeks after treatment. (G) Percentage of lung area affected by inflammatory lesions relative to total lung area, determined by morphometric analysis. Reproduced with permission. [251] Copyright 2020, MDPI.

Liang et al. [251] investigated the immunoregulatory and antimicrobial properties of bovine neutrophil β‐defensin‐5 (B5)‐loaded PLGA nanoparticles (B5‐NPs) in a murine model challenged with *M. bovis* (Figure 10A). B5 is a naturally occurring AMP with immunomodulatory properties that was previously shown to reduce mycobacterial viability in vitro. To encapsulate B5, the authors employed a double emulsion (water–oil–water) solvent evaporation method, a well‐established in situ technique that enables efficient incorporation of hydrophilic proteins within polymeric matrices. This method yielded smooth, spherical nanoparticles with a mean diameter of 206.6 ± 26.6 nm, a polydispersity index of 0.16, and a ZP of –27.1 ± 1.5 mV (Figure 10B). The encapsulation efficiency reached 85.5 ± 2.5%, indicating a highly efficient loading process. In vitro release studies under physiological conditions revealed a biphasic pattern, with initial burst release of 18% within 2 h followed by sustained release, reaching 67% of B5 by the end of day 7 (Figure 10C). Importantly, B5 released from the nanoparticles retained its structural integrity, as confirmed by SDS‒PAGE and Western blotting. In vitro studies revealed that B5‐NPs stimulated J774A.1 macrophages to secrete the proinflammatory cytokines TNF‐α and IL‐10 more robustly than free B5 or control NPs did. For in vivo analysis, a challenge test was conducted: Mice were first immunized with BCG and then intranasally boosted three times with B5 or B5‐NPs before being challenged with *M. bovis* (∼1000 CFU intranasally). Histopathological evaluation using hematoxylin–eosin and acid‐fast staining demonstrated a marked reduction in pulmonary inflammatory lesions and mycobacterial presence in the B5‐NPs group compared with controls (Figure 10D). Four weeks post challenge, the mice immunized with B5‐NPs presented markedly reduced bacterial loads in the lungs (Figure 10E) and spleen (Figure 10F), diminished pulmonary inflammation (Figure 10G), elevated TNF‐α and IgA levels, and increased percentages of CD4^+^ and CD8^+^ T cells in the spleen. These effects were superior to those observed with free B5. While free B5 displayed some therapeutic effects in combination with RIF, the PLGA‐based nanoparticles demonstrated superior immunogenicity, reduced TNF‐α hypersecretion and lung pathologing, positioning it as a promising adjuvant and delivery nanosystem for peptide‐based TB vaccines.

**FIGURE 11:**
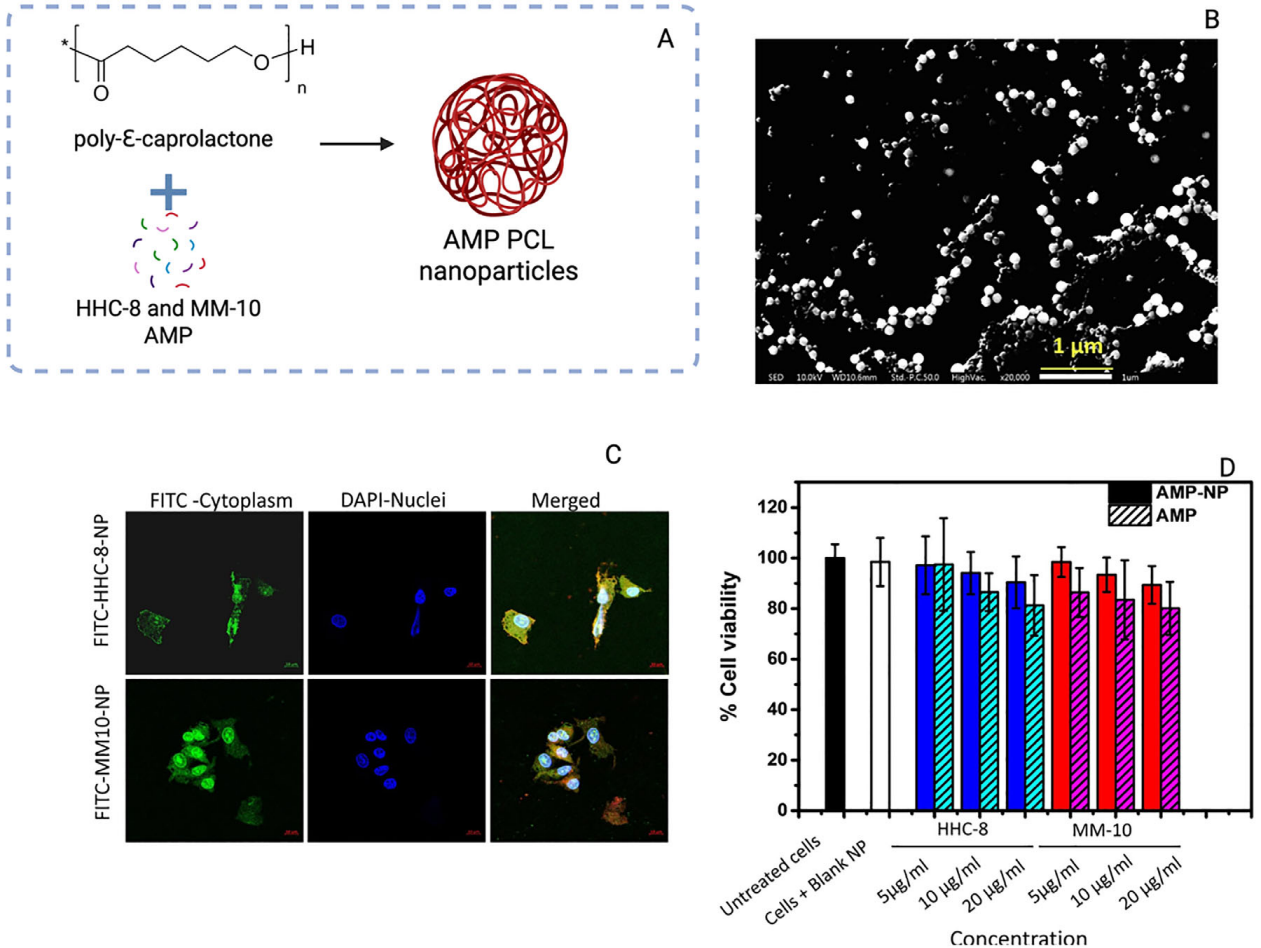
Development and evaluation of AMP‐loaded poly(ε‐caprolactone) (PCL) nanoparticles. (A) Schematic representation of nanoparticle preparation: HHC‐8 and MM‐10 AMPs were encapsulated into PCL‐based nanoparticles via nanoprecipitation. (B) Scanning electron microscopy (SEM) image of AMP‐loaded PCL nanoparticles showing a spherical morphology and uniform size distribution. Scale bar = 1 µm. (C) Confocal microscopy images of macrophages treated with FITC‐labeled AMP‐loaded nanoparticles (FITC‐HHC‐8‐NPs and FITC‐MM10‐NPs). Green fluorescence corresponds to the cytoplasmic FITC signal, and blue fluorescence indicates DAPI‐stained nuclei. Merged images showing successful cellular uptake of the nanoparticles. Scale bars = 20 µm. (D) In vitro cytotoxicity of free AMPs and AMP‐loaded nanoparticles (AMP‐NPs) at different concentrations (5, 10, and 20 µg/mL) assessed in RAW 264.7 macrophages. Reproduced with permission [252]. Copyright 2021, Elsevier.

Sharma et al. [252] designed poly(ε‐caprolactone) (PCL) nanoparticles loaded with synthetic peptides HHC‐8 [KIWWWWRKR] and MM‐10 [MLLKKLLKKM] (Figure 11A). The peptides, both cationic and α‐helical, were rationally designed and synthesized via Fmoc‐based solid‐phase peptide synthesis, aiming to optimize their antimicrobial potency against *Mtb* and *M. smegmatis*. HHC‐8 has been previously shown to interact directly with bacterial membranes through tryptophan‐rich domains, whereas MM‐10 disrupts lipid bilayers by forming stable pores, with documented synergism when combined with RIF. Nanoparticles were produced via a water‐in‐oil‐in‐water (W/O/W) double emulsion solvent evaporation technique, which is a well‐established in situ encapsulation technique that allows peptides to be embedded within the PCL matrix during nanoprecipitation. PCL‐HHC‐8 and PCL‐MM‐10 nanoparticles had average diameters of 376.5 and 289.9 nm, ZP values of 26.2 and 15.8 mV, and EE values of ∼18.9% and ∼21.1%, respectively. SEM images confirmed the spherical shape and uniform distribution of both samples (Figure 11B). The release assay of the nanoparticles containing the peptides was performed in simulated lung medium (PBS, pH 7.4), which demonstrated a prolonged release profile, with release rates of 65 and 43% for PCL‐MM‐10 and PCL‐HHC‐8, respectively, within 24 h. Confocal microscopy analysis revealed significant cellular uptake of both nanosystems in mouse macrophages (RAW 264.7) (Figure 11C). Cytocompatibility assays revealed that free HHC‐8 (20 µg/mL) reduced macrophage viability to ∼85%, whereas PCL‐HHC‐8 maintained viability at ∼95%. Similarly, free MM‐10 reduced viability to ∼81%, whereas PCL‐MM‐10 preserved it at ∼91% (Figure 11D). PCL‐HHC‐8 and PCL‐MM10 NPs showed superior antimicrobial activity, with MIC values of 9 and 18.75 µg/mL for *Mtb* and *M. smegmatis*, respectively, while the free peptides had MICs of >75 µg/mL for both strains. Notably, coadministration of the nanosystem with RIF yielded strong synergistic effects, with fractional inhibitory concentration indices (FICIs) ≤ 0.09. Additionally, combining PCL‐HHC‐8 and PCL‐MM‐10 produced a FICI of ∼0.53, indicating additive‐to‐synergistic activity. This codelivery strategy significantly enhances antimycobacterial activity and offers a potential combination therapy for TB involving AMPs and first‐line antibiotics.

**FIGURE 12:**
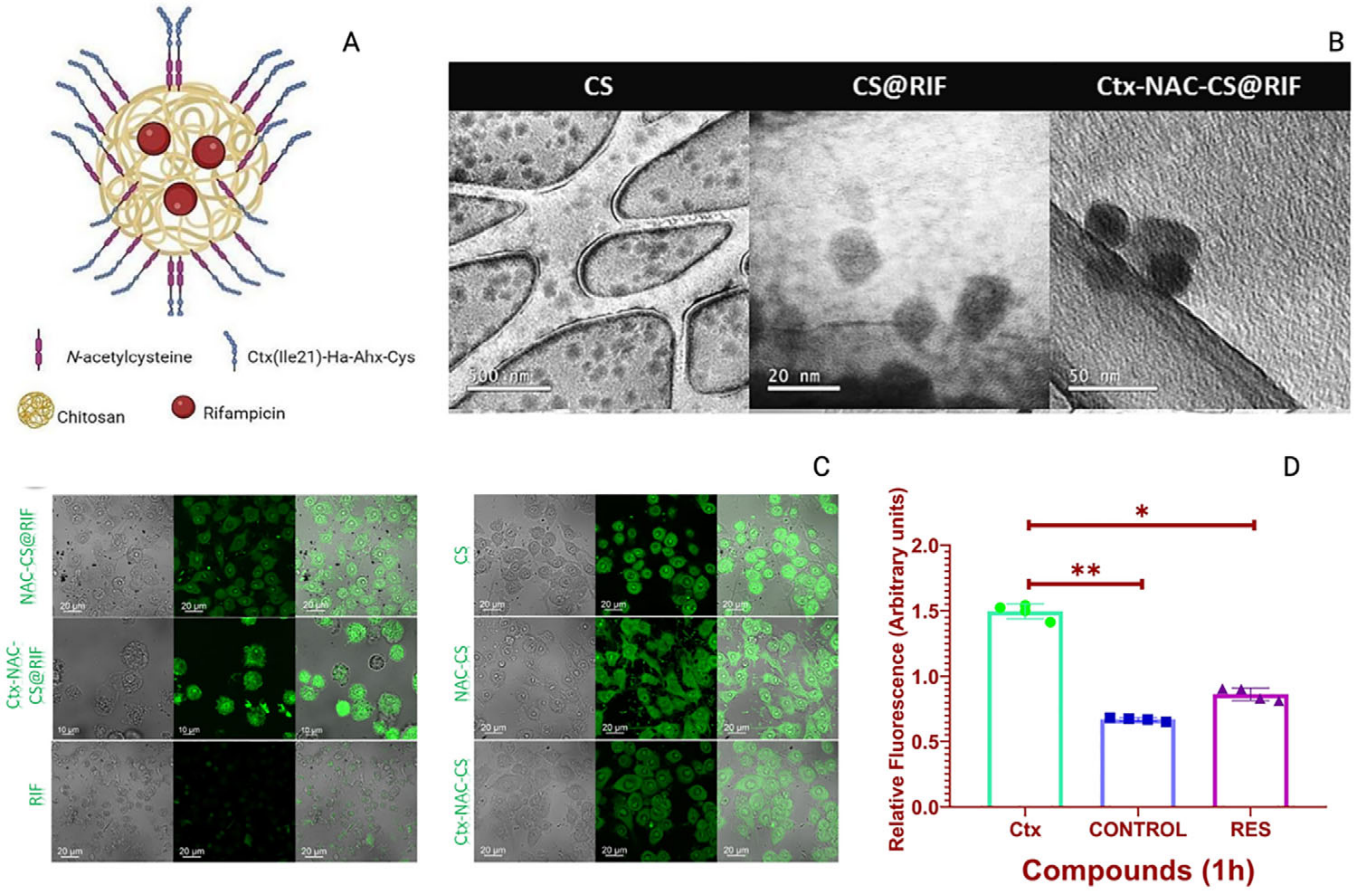
Design, characterization, and cellular interaction of multifunctional chitosan‐based nanoparticles coloaded with RIF (RIF) and functionalized with Ctx peptide and N‐acetylcysteine (NAC). (A) Schematic representation of the nanoparticle structure composed of chitosan, RIF, N‐acetylcysteine (NAC), and the Ctx(Ile21)‐Ha‐Ahx‐Cys peptide. (B) TEM images showing the morphological evolution from plain chitosan nanoparticles (CSs) to RIF‐loaded chitosan (CS@RIF) and then to dual‐functionalized systems (Ctx‐NAC‐CS@RIF). (C) Cellular uptake of formulations evaluated by fluorescence microscopy using FITC‐labeled systems. Enhanced intracellular fluorescence is observed for Ctx‐functionalized formulations (Ctx‐MAC‐CS@RIF and NAC‐CS@RIF) compared with nonfunctionalized controls. (D) Accumulation of ethidium bromide in *Mtb* H_37_Rv after 1 h treatment, showing that the Ctx(Ile21)‐Ha‐Ahx‐Cys peptide significantly inhibits efflux pump activity, as indicated by increased intracellular fluorescence compared to controls. The data are presented as the means ± SDs; *p < 0.05, **p < 0.01. Reproduced with permission [143]. Copyright 2024, Elsevier.

The modification of CS with N‐acetylcysteine (NAC) may favor antibacterial activity because of its ability to inhibit the formation of biofilms from respiratory tract infections. In a recent study, Primo et al. [143] explored Ctx (Ile 21)‐Ha‐Ahx‐Cys peptide‐grafted N‐acetylcysteine‐CS (NAC‐CS) nanoparticles through disulfide bond formation to revitalize RIF activity against clinical isolates of *Mtb*, including multidrug‐ and extensively drug‐resistant (MDR/XDR) strains (Figure 12A). The AMP Ctx, a synthetic analog of a frog‐derived antimicrobial peptide, has an amphipathic α‐helical structure and acts by forming pores in the bacterial membrane, promoting rapid cell lysis—even in drug‐resistant strains. NAC, on the other hand, is known for its antibiofilm and mucolytic properties, enhancing nanoparticle mucoadhesion and stability under respiratory conditions. The NAC‐CS biopolymer was first synthesized through amide coupling between NAC and CS via EDC/NHS chemistry, followed by an oxidative disulfide linkage with the thiolated AMP (Ctx). Then, RIF‐loaded nanoparticles (Ctx‐NAC‐CS@RIF) were synthesized via the ionotropic gelation method via the cross‐linking agent sodium tripolyphosphate (TPP). The NPs presented an average size of 123.9 and a zeta potential of 37 mV, which remained positive after functionalization with the peptide and incorporation of the drug. The spherical morphology was confirmed via TEM (Figure 12B). The antimicrobial activity of NP8 was tested against *Mtb* H_37_Rv and two drug‐resistant clinical isolates (CF169/XDR and CF110/MDR). Ctx‐NAC‐CS@RIF showed superior efficacy, with an MIC of approximately 0.97 µg/mL against the H_37_Rv strain and CF 169‐ and CF 110‐resistant clinical isolates, whereas the free drug exhibited MICs of 0.5, >25 and 16.29 µg/mL, respectively. Neither NAC‐CS@RIF (without AMP) nor Ctx‐NAC‐CS (without RIF) showed activity against resistant strains, and free Ctx alone had limited efficacy (MIC ∼7.6 µg/mL for Ctx‐Cys; ∼14 µg/mL for wild‐type Ctx). Furthermore, Ctx‐NAC‐CS@RIF showed no toxicity to macrophages or fibroblasts, with no reduction in viability at concentrations up to 100 µg/mL. Compared with free RIF, the uptake of FITC‐labeled Ctx‐NAC‐CS@RIF by macrophages was significantly greater, suggesting that AMP grafting facilitates cellular internalization (Figure 12C). Functional assays demonstrated that the Ctx(Ile21)‐Ha‐Ahx‐Cys peptide can inhibit the efflux pump activity of *Mtb*, as evidenced by a significant increase in intracellular accumulation of ethidium bromide in treated bacteria (Figure 12D). This finding indicates that, beyond its membrane‐disrupting activity, the AMP can block efflux‐mediated drug resistance, further sensitizing the bacteria to rifampicin. This dual action—membrane disruption and efflux pump inhibition—provides a powerful multifaceted strategy for overcoming resistance. The association of Ctx with NAC‐CS NPs A represents a potential strategy that leads to multivalent attack, disrupting bacterial membranes while enhancing drug retention in infected macrophages, highlighting Ctx‐NAC‐CS as a smart multifunctional nanocarrier for TB therapy.

**FIGURE 13:**
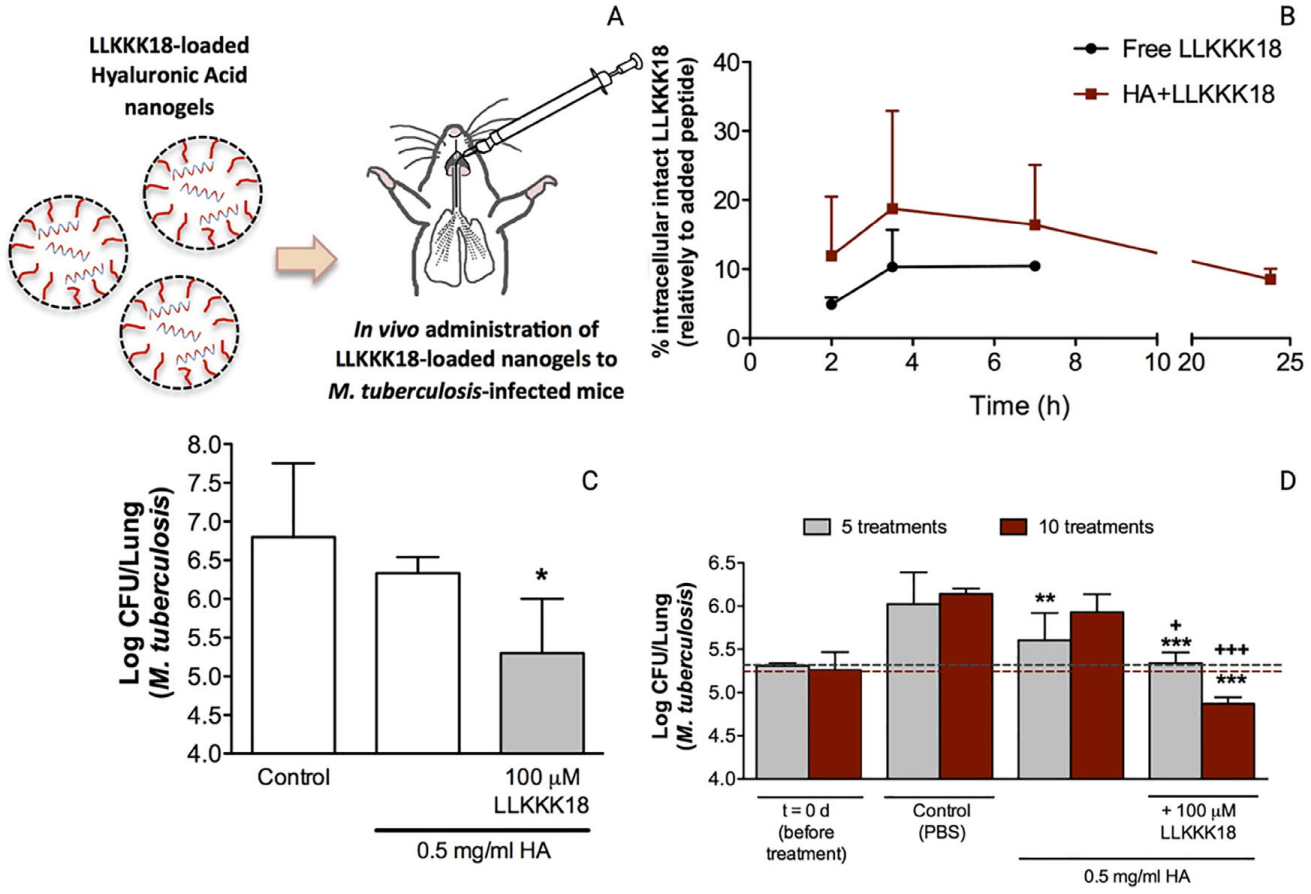
In vivo evaluation of the effects of LLKKK18‐loaded hyaluronic acid (HA) nanogels against *Mtb* infection. (A) Schematic representation of HA nanogels encapsulating the cationic AMP LLKKK18 and their intranasal administration in *Mtb*‐infected mice. (B) Intracellular levels of intact LLKKK18 peptide in macrophages over time, comparing free LLKKK18 and HA‐encapsulated LLKKK18. The HA nanogels improved peptide stability and intracellular retention. (C) Bacterial load in mouse lungs (expressed as log CFU/lung) after treatment with LLKKK18‐loaded nanogels, showing a significant reduction in bacterial burden compared with that of the untreated control group. (D) Comparative efficacy of five versus ten intranasal treatments with LLKKK18‐loaded nanogels, demonstrating a dose‐dependent reduction in the pulmonary *Mtb* load. Reproduced with permission [253]. Copyright 2016, Elsevier.

Hyaluronic acid (HA) has also been explored as a potential strategy for the treatment of TB infections because of its ability to recognize CD44 receptors and improve cellular internalization in infected macrophages. Silva et al. [253] developed self‐assembling hyaluronic acid nanogels to deliver the cationic antimicrobial peptide LLKKK18 (KEFKRIVKRIKKFLRKLV) (LK18‐HANG) (Figure 13A), a synthetic analog of LL‐37—designed to enhance membrane disruption through increased cationicity and hydrophobicity. LLKKK18 exhibits broad‐spectrum antimicrobial activity by permeabilizing bacterial membranes but suffers from high cytotoxicity and rapid degradation under physiological conditions. To overcome these drawbacks and improve targeting infected macrophages, the authors encapsulated the peptide into hydrophobized HA nanogels, leveraging HA's affinity for CD44 receptors, which are upregulated on activated macrophages during infection. The nanogels were synthesized by chemically grafting 11‐amino‐1‐undecanethiol (AT) to HA via EDC/NHS coupling, which induced amphiphilicity and self‐assembly in aqueous media. Peptide encapsulation was performed via electrostatic interaction, and the LLKKK18 solution was incubated with the nanogels under gentle rotation for 24 h. The resulting LK18‐HANGs exhibited high encapsulation efficiency (∼70%), hydrodynamic diameter of ∼533 nm, monomodal size distribution (PDI ∼0.1), and zeta potential near neutrality (2.4 mV) due to charge neutralization by the highly cationic peptide. In vitro cytotoxicity assays demonstrated that free LLKKK18 at 100 µM reduced macrophage viability by ∼90%, whereas LK18‐HANGs preserved ∼100% viability at the same concentration, confirming the protective role of encapsulation. Importantly, LK18‐HANGs reduced intracellular *M. avium* CFU counts by up to 4.4 log, whereas free peptide and blank nanogels had no antimycobacterial effect, highlighting the necessity of peptide encapsulation for intracellular efficacy (Figure 13B). In vivo, mice chronically infected with *M. avium* strains 2447 and 25291 were treated via intratracheal administration of LK18‐HANGs (100 µm, five doses). The treatment reduced pulmonary bacterial loads by ∼1 log (2447) and ∼0.3 log (25291). In comparison, mice treated with free LLKKK18 or blank HANGs presented no significant reduction in CFUs, demonstrating that only the peptide‐loaded nanogels exerted therapeutic effects in vivo (Figure 13C,D). Confocal microscopy and MALDI‐TOF MS confirmed the presence of intracellular peptide localization, with partial colocalization near phagosomal compartments. Moreover, LK18‐HANGs modulated the inflammatory response in *Mtb*‐infected macrophages by reducing TNF‐α and IL‐6 secretion, suggesting dual antimicrobial and immunomodulatory actions [253]. These findings indicate the potential of HA nanogels to increase the intracellular activity of peptides in treatment. of pulmonary infections.

#### 5.2.2 Inorganic Nanoparticles

Inorganic nanoparticles stand out from their organic counterparts by offering exceptional structural robustness, high surface‐to‐volume ratios, and intrinsic physicochemical properties—such as optical, catalytic, and magnetic behaviors—that can be exploited for therapeutic and diagnostic purposes [22]. Unlike polymeric or lipid‐based systems, which rely predominantly on passive encapsulation and surface modification, inorganic platforms often exhibit inherent antimicrobial activity (e.g., silver or zinc oxide) or serve as theranostic agents (e.g., gold or iron oxide) [254], reducing the need for additional functional components. Notably, silver nanoparticles (AgNPs) have been widely studied for their ability to disrupt bacterial membranes and generate reactive oxygen species (ROS), which function both as carriers and direct antimicrobial agents [255, 256, 257, 258]. Although they are inert, gold nanoparticles (AuNPs) can be easily functionalized with peptides or ligands via thiol chemistry and offer unique plasmonic properties for tracking or triggered release [259, 260, 261].

MSNs, on the other hand, possess high surface areas (typically >500 m^2^/g), uniform and tunable pore sizes (2–50 nm), and large loading capacities for peptide encapsulation [262, 263, 264, 265]. Their surface can be easily functionalized with targeting ligands, polymers, or pH‐sensitive gates, promoting the design of stimuli‐responsive nanocarriers to target and deliver drugs in infected tissues or intracellular compartments [266, 267]. MSNs have demonstrated remarkable potential for intracellular AMP delivery, particularly due to their ability to penetrate granulomas and deliver peptides directly into infected macrophages [268]. Although MSNs have been widely studied for their high surface area, tunable porosity, and ease of functionalization, their in vivo fate depends on multiple factors, including particle size, surface chemistry, and administration route. While MSNs can undergo slow biodegradation and be excreted via renal and fecal routes [269, 270], comprehensive safety evaluation and dose optimization remain essential steps to ensure their clinical viability, an aspect that applies broadly across all nanoparticle platforms. [271, 272]. Despite these limitations, inorganic nanoparticles offer unique physicochemical properties that, when carefully engineered, can enhance AMP protection, targeting, and therapeutic efficacy in TB therapy [264]. Particular attention is required for metallic nanoparticles such as gold and silver, which, owing to their ultrasmall size and high surface reactivity, pose additional concerns regarding their biodistribution, long‐term accumulation, and toxicity [273]. To address these challenges, such systems are frequently coated with biocompatible materials such as silica, polymers, or lipids or integrated into hybrid platforms such as carbon nanotubes, which help modulate their biological interactions and improve their translational potential.

Beitzinger et al. [274] used dendritic mesoporous silica nanoparticles (DMSNs) as carriers for the intracellular delivery of NapFab, a synthetic AMP derived from Napsin A‐derived AMP that was initially identified from bronchoalveolar lavage fluid and subsequently optimized to enhance its solubility and cationic character. In addition to its antimicrobial activity, NapFab possesses intrinsic cell‐penetrating properties, making it particularly suitable for targeting *Mtb* residing within host macrophages. The DMSNs exhibited a uniform spherical morphology with an average diameter of approximately 163 nm, a specific surface area of 554 m^2^/g, and a pore diameter of ∼4.7 nm. NapFab was loaded into DMSN via aqueous solution adsorption, resulting in a high loading capacity of ∼27%. DLS analyses revealed that NapFab@DMSN had an average diameter of 179.2 nm and a ZP of –24.9 mV when dispersed in serum‐free macrophage medium. Considering that blank DMSNs presented a zeta potential of –25.7 mV under the same conditions, the minimal shift suggests that NapFab was predominantly loaded inside the mesopores rather than adsorbed onto the external surface, thereby maintaining colloidal stability. In vitro assays using infected murine macrophages revealed that free NapFab at 100 µm inhibited intracellular *Mtb* growth by approximately 40%, whereas NapFab@DMSN at only 5 µm achieved ∼80% inhibition. Furthermore, confocal microscopy revealed that NapFab@DMSN localized in proximity to phagosomal membranes, enabling gradual cytosolic release of NapFab. The authors assessed the integrity of the cell membrane via flow cytometry, and the results revealed that neither the NapFab@DMSN nor free NapFab caused membrane disruption. The safety profile of NapFab@DMSN and free NapFab was confirmed through an in vivo toxicity model using zebrafish embryos. Lattice light sheet fluorescence microscopy revealed that NapFab was released intracellularly from the DMSN within primary human macrophages. These results demonstrate that DMSNs substantially enhance the intracellular delivery, stability, and efficacy of NapFab against *Mtb*, even at lower concentrations, by protecting them from premature degradation and ensuring their release in infected cells.

**FIGURE 14:**
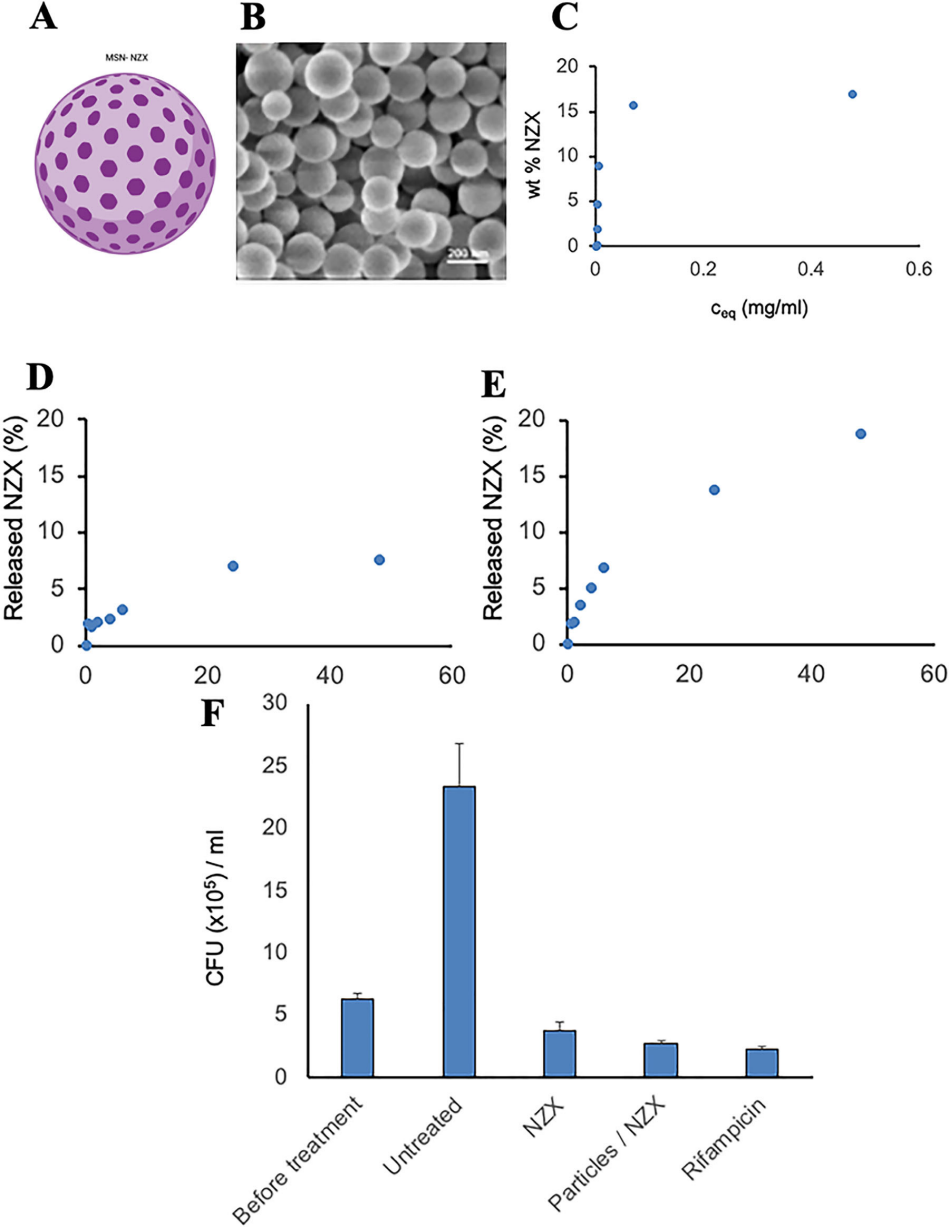
(A) Schematic representation of NZX‐loaded MSNs; (B) Scanning electron microscopy image of MSNs; (C) absorption isotherm of the NZX peptide. Release profile of NZX‐loaded MSNs in (D) PBS and (E) simulated lung fluid. (F) Evaluation of MSN efficacy in an in vivo murine model of TB [275]. Reprinted from an open‐access article by PLOS Copyright 2019.

Tenland et al. [275] designed and evaluated MSN for the intracellular delivery of the antimicrobial peptide NZX, a defensin‐like molecule cyclized by three disulfide bridges (Figure 14A). The MSNs had a mean diameter of approximately 200 nm, a BET surface area of 1070 m^2^/g, a pore diameter of ∼3 nm, a pore volume of 0.8 mL/g, and a zeta potential of −26 mV—parameters compatible with high peptide loading and rapid internalization by macrophages. Scanning electron microscopy revealed homogeneous, submicrometer‐sized spherical particles (∼200 nm) (Figure 14B). The adsorption isotherm demonstrated strong electrostatic interactions between cationic NZX and the anionic silica matrix, reaching a plateau of ∼17% w/w loading, indicative of predominant adsorption within the pore network (Figure 14C). Release studies showed that MSNs released 7.6% of the loaded NZX after 48 h in PBS (Figure 14D). In simulated lung fluid (SLF, pH 7.4) containing 0.02% w/v DPPC, the 48 h release increased to 18.8%, attributed to lipid competition for silica adsorption sites (Figure 14E). REMA assays showed an identical MIC of 3.2 µm for free NZX and MSN‐loaded NZX, indicating that the loading process did not reduce its antimycobacterial potency. In the time‐kill assay, NZX released from MSNs and freshly prepared NZX at 3.2 µm exhibited the same inhibition kinetics against *M. bovis* BCG. Uptake studies with Atto488‐labeled MSNs demonstrated rapid, concentration‐dependent internalization: at 300 µg/mL and 30 min, most primary macrophages had internalized particles, compared to ∼80% for THP‐1 monocytes; at 25 µg/mL, uptake was ∼80% and 40%, respectively. TEM revealed storage in vesicle‐like structures with progressive degradation by 72 h, while confocal microscopy confirmed internalization even at 5 µg/mL. Cytotoxicity assays (ATPlite/MTT) indicated dose‐dependent but manageable toxicity at therapeutic ranges. In intracellular infection assays, MSN/NZX achieved significantly greater mycobacterial inhibition compared to free NZX. In the murine pulmonary TB model with *Mtb* H_37_Rv, the implanted dose at 48 h was 520 ± 32 CFU, increasing to 6.3 × 10^5^ CFU/mL after 14 days. Following five intratracheal doses (NZX 0.83 mg; MSN/NZX containing 0.83 mg NZX in 5 mg MSN; or rifampicin 20 mg/kg), CFU reductions compared to untreated controls were 84% (NZX), 88% (MSN/NZX), and 90% (rifampicin), with no significant differences among the active treatments (Figure 14F). The study demonstrates that MSNs can maintain the antimicrobial activity of NZX while increasing its accumulation in host cells. This is particularly relevant as it allows a gradual release at the site of infection. Therefore, the same strategy could also be applied to other antimicrobial peptides, offering opportunities for targeted pulmonary treatments against tuberculosis and other intracellular infections.

Mohanty et al. [276] designed two biogenic silver nanoparticles (AgNPs) using plant‐based (*Alstonia macrophylla*, NP‐1) and fungal‐based (*Trichoderma* sp., NP‐2) biomasses as reducing and stabilizing agents. The synthesis was confirmed by the appearance of a brown color and Ultraviolet‐Visible (UV‐Vis) absorbance at 420 nm, followed by morphological characterization. TEM revealed spherical nanoparticles with average sizes of ∼50 nm (NP‐1) and ∼100 nm (NP‐2), whereas dynamic light scattering (DLS) revealed hydrodynamic diameters of 177 and 97.65 nm, respectively. Interestingly, NP‐1 displayed a negative ZP (–2.7 mV), whereas NP‐2 was positively charged (+13 mV), likely due to differences in the natures of the capping agents derived from each biomass source. The cationic AMPs NK‐2 and LLKKK‐18 were subsequently noncovalently associated with the AgNPs via adsorption. NK‐2 is a synthetic derivative of the cytolytic protein NK‐lysin and exhibits broad‐spectrum antimicrobial activity. LLKKK‐18 is a rationally designed variant of human LL‐37 engineered to increase the cationic charge and activity against mycobacteria. In antimicrobial assays against *M. smegmatis*, both AgNP formulations loaded with NK‐2 achieved >90% bacterial killing at 0.5 ppm (NP‐1 or NP‐2) plus 7 µg/mL NK‐2, significantly outperforming NK‐2 alone, which caused only moderate inhibition under the same conditions. In contrast, LLKKK‐18 alone (1 µg/mL) produced only moderate inhibition, but when LLKKK‐18 was combined with 0.5 ppm NP‐2, it resulted in 89% bacterial reduction, indicating an additive effect exclusive to the NP‐2 formulation. This selectivity may be related to the positive surface charge of NP‐2, which enhances electrostatic interactions with the cationic peptide. No synergistic effect was observed between LLKKK‐18 and NP‐1. The formulations were evaluated in infected murine macrophages. LLKKK‐18–NP‐1 reduced the number of intracellular CFUs of *M. smegmatis* by ∼65%, and NK‐2–NP‐2 achieved ∼52% killing, indicating greater efficacy than either component alone. Importantly, these combinations were noncytotoxic at doses up to 5 ppm and did not induce genotoxicity, as confirmed by MTT, trypan blue exclusion, comet, and micronucleus assays. Fluorescence microscopy and flow cytometry confirmed the efficient uptake of AgNPs by macrophages, particularly for NP‐2, which showed 15.2% uptake versus only 1.8% for NP‐1 after 12 h. Notably, all AgNP–peptide formulations retained colloidal and structural stability for at least seven days under physiological conditions, a critical requirement for systemic or pulmonary delivery. Together, these findings underscore the potential of biogenic silver nanoparticles as stable and synergistic carriers for AMPs in targeting intracellular mycobacterial infections.

### 5.3 Pharmacokinetics, Biodistribution, and Long‐Term Safety Considerations

Effective nanocarrier delivery for TB should be evaluated by whether the formulation can access heterogeneous lung lesions and their distinct microenvironments, rather than being judged primarily by plasma exposure or average lung concentrations [208]. This distinction matters because drug penetration is often uneven across lesion compartments, a pattern that has been associated with differences in sterilizing activity and can open periods of functional monotherapy that promote resistance [279]. Consistent with this, lesion‐resolved analyses of human lungs support quantitative, compartment‐specific exposure as a central translational endpoint when advancing new delivery platforms, including nanoformulated AMPs [280].

Within this framework, in vivo performance is usually governed less by in vitro stability than by transport barriers and clearance kinetics, so improvements should be supported by time‐resolved measurements at the relevant pulmonary sites [196]. An additional complication is that nanoparticles rapidly acquire a biomolecular corona in biological fluids, meaning that the in vivo identity that dictates distribution and cellular uptake can diverge substantially from the pristine formulation characterized in buffer [281].

These considerations become even more consequential for inhaled delivery, where aerosol properties that determine regional deposition, such as aerodynamic diameter, and fine particle fraction, need to be reported and tied to the intended target region of the respiratory tract [282]. After deposition, nanoparticles may be retained in the lung, taken up by alveolar macrophages, and in some cases translocated to secondary organs, making both pulmonary residence and systemic spillover integral to biodistribution assessment [283]. Because mucociliary clearance does not necessarily slow in a predictable way with nanosizing or surface charge, claims about extended lung residence should be supported by direct clearance measurements rather than inferred from particle design alone [284]. At the same time, pulmonary surfactant represents the first major interface for inhaled particulates and can modulate dispersion and downstream biological responses, which argues for evaluating surfactant interactions during formulation development [285]. Moreover, airway disease can shift deposition patterns and, with them, exposure and risk profiles; accordingly, TB‐relevant pulmonary models should incorporate regional distribution measurements instead of extrapolating from deposition in healthy lungs [286].

Finally, the safety package must extend beyond short‐term cytotoxicity screening, since in vitro assays can be affected by methodological limitations and nanomaterial‐related interferences and may overlook immune‐relevant dysfunction [287]. Because engineered nanomaterials can elicit distinct immune responses as a function of their physicochemical properties, routine assessment of macrophage activation and inflammatory signaling is warranted alongside viability metrics [288]. This is particularly important for inorganic systems, which may undergo progressive in vivo transformation that influences persistence and organ retention, making long‐term fate measurements an essential complement to repeated‐dose biodistribution studies [289]. In line with this, extended safety evaluation is especially critical for non‐biodegradable inorganic carriers, where subchronic or chronic dosing can reveal liabilities that are not apparent from acute tolerability or short exposure windows [290].

### 5.4 Manufacturing, Cost, and Implementation in High‐Burden Settings

TB incidence and mortality remain concentrated in low‐ and middle‐income countries, so AMP nanocarrier approaches need to be assessed against the affordability, procurement, and delivery constraints of national TB programmes [291]. Modeling studies of reduced international donor support indicate that even moderate funding contractions can increase TB morbidity and mortality, which limits the headroom for higher‐cost modalities at scale [292].

On the active‐ingredient side, therapeutic peptides are commonly produced by solid‐phase synthesis, where the number of coupling cycles and the extent of GMP‐grade purification drive effort and cost as sequence length and chemical complexity increase [293]. Process mass‐intensity analyses show that post‐cleavage workup, purification, and final recovery operations account for a substantial fraction of material use, linking cost‐of‐goods to downstream unit operations rather than to synthesis alone [294]. Reviews of peptide therapeutics recommend integrating manufacturability and analytical release feasibility during candidate selection, including sequence and chemistry choices that support robust specifications and quality control at production scale [295].

For the carrier component, experience drawn from clinically translated nanoparticle medicines indicates that attribute control, batch‐to‐batch comparability, and validated specifications are central to regulatory approval and can dominate development resources [296]. Manufacturing studies report that microfluidic and continuous processing can improve reproducibility by tightening control over particle attributes during scale‐up of complex nanomedicines [297]. Quality‐by‐design frameworks for nanotechnology‐based drug products recommend early definition of critical quality attributes and their linkage to critical process parameters to support technology transfer and reduce batch failure risk [298]. For pulmonary TB, inhalable dry powder approaches have been discussed as deployable options in endemic settings because they can improve storage stability and reduce reliance on cold‐chain logistics relative to some liquid dispersions [299]. The deliver framework places manufacturability, regulatory strategy, and access planning alongside efficacy during development to maintain compatibility with health‐system constraints in high‐burden countries [300].

## 6 Conclusions and Perspectives

Conventional TB therapy faces significant and multifactorial challenges, including the biological complexity of *Mtb*, the emergence of MDR and XDR strains, and the prolonged duration of current therapeutic regimens. These obstacles are further exacerbated by the low bioavailability of drugs, rapid degradation of therapeutic agents, high treatment costs, and persistent barriers to patient adherence—factors that are particularly pronounced in low‐income and middle‐income countries. In this context, AMPs have emerged as promising therapeutic candidates because of their broad‐spectrum activity and low tendency to induce bacterial resistance. However, the clinical application of AMPs remains limited because of their susceptibility to proteolytic degradation and poor in vivo stability. Nanotechnology has shown significant potential to overcome these limitations, as nanocarrier systems can protect AMPs, increase their bioavailability, and facilitate their targeted release to infection sites, such as macrophages and granulomas. Notably, the functionalization strategies of nanocarriers can further enhance therapeutic selectivity and efficacy; however, most published studies remain at in vitro or proof‐of‐concept stages, and experimental evidence specifically targeting *Mtb* remains scarce. In parallel, in silico methodologies—such as molecular docking and molecular dynamics simulations—have accelerated the rational design and optimization of AMPs, enabling the identification of candidates with increased specificity and potency against *Mtb*. These computational approaches help shorten drug development timelines and reduce associated costs; nonetheless, experimental validation remains essential to translate these findings into effective therapies. Despite these advances, multiple challenges persist. There is a clear need to expand preclinical and clinical studies to rigorously evaluate the efficacy and safety of AMP‐based nanotherapeutics, particularly in animal models and human populations. It is also necessary to assess long‐term impacts, such as the potential accumulation of nanocarriers in specific organs, thoroughly. Furthermore, the scalability and cost‐effective production of these advanced systems represent major hurdles for their global implementation. Future research should focus on optimizing AMP delivery systems and exploring alternative administration routes, such as inhalation, which would allow more direct and efficient action at pulmonary sites. It will also be crucial to develop computational platforms integrated with artificial intelligence for the precise design of next‐generation AMPs. The establishment of robust regulatory frameworks and the promotion of international collaboration will be essential to ensure that innovations in nanotechnology and peptide‐based therapies translate into tangible and equitable benefits for global public health. Upholding ethics and equity as guiding principles must remain a fundamental priority throughout the development and implementation of these technologies.

## Author Contributions

**Christian S. Carnero Canales**: writing – review & editing, writing – original draft, validation, investigation, conceptualization. **Jessica Ingrid Marquez Cazorla** and **Renzo Marianito Marquez Cazorla**: writing – original draft, visualization, validation, investigation, and formal analysis. **Aline Martins dos Santos, Jonatas Lobato Duarte, Letícia Oliveira Catarin Nunes, Túlio Custódio Reis**, and **Lara Cerazi Salvador**: writing – original draft, investigation, formal analysis. **Norival Alves Santos‐Filho** and **Rafael Miguel Sábio**: writing – review & editing, visualization, validation, supervision. **Hélder A. Santos**: writing – review & editing, validation, supervision, resources, project administration, funding acquisition, conceptualization. **Fernando Rogério Pavan**: writing – review & editing, visualization, validation, supervision, resources, project administration, funding acquisition, conceptualization.

## Conflicts of Interest

The authors declare no conflict of interest.

## Declaration of Generative AI and AI‐Assisted Technologies in the Writing Process

During the preparation of this work, the authors used ChatGPT and Curie to increase the readability and language of the manuscript. After using these tools and services, the authors thoroughly reviewed and edited the content as needed and took full responsibility for the final version of the published article.

## Data Availability

No new data were generated or analyzed in this review. All data discussed are available in the cited literature.
